# Convergence of Age Differences in Risk Preference, Impulsivity, and Self-Control: A Multiverse Analysis

**DOI:** 10.1093/geronb/gbae092

**Published:** 2024-05-23

**Authors:** Loreen Tisdall, Renato Frey, Dirk U Wulff, David Kellen, Rui Mata

**Affiliations:** Faculty of Psychology, Center for Cognitive and Decision Sciences, University of Basel, Basel, Switzerland; Cognitive and Behavioral Decision Research, University of Zurich, Zurich, Switzerland; Faculty of Psychology, Center for Cognitive and Decision Sciences, University of Basel, Basel, Switzerland; Center for Adaptive Rationality, Max Planck Institute for Human Development, Berlin, Germany; Department of Psychology, College of Arts and Sciences, Syracuse University, Syracuse, New York, USA; Faculty of Psychology, Center for Cognitive and Decision Sciences, University of Basel, Basel, Switzerland; (Psychological Sciences Section)

**Keywords:** Biology, Cross-sectional life span trajectories, Revealed preferences, Risk taking, Stated preferences

## Abstract

**Objectives:**

Numerous theories exist regarding age differences in risk preference and related constructs, yet many of them offer conflicting predictions and fail to consider convergence between measurement modalities or constructs. To pave the way for conceptual clarification and theoretical refinement, in this preregistered study we aimed to comprehensively examine age effects on risk preference, impulsivity, and self-control using different measurement modalities, and to assess their convergence.

**Methods:**

We collected a large battery of self-report, informant report, behavioral, hormone, and neuroimaging measures from a cross-sectional sample of 148 (55% female) healthy human participants between 16 and 81 years (mean age = 46 years, standard deviation [*SD*] = 19). We used an extended sample of 182 participants (54% female, mean age = 46 years, *SD* = 19) for robustness checks concerning the results from self-reports, informant reports, and behavioral measures. For our main analysis, we performed specification curve analyses to visualize and estimate the convergence between the different modalities and constructs.

**Results:**

Our multiverse analysis approach revealed convergent results for risk preference, impulsivity, and self-control from self- and informant reports, suggesting a negative effect of age. For behavioral, hormonal, and neuroimaging outcomes, age effects were mostly absent.

**Discussion:**

Our findings call for conceptual clarification and improved operationalization to capture the putative mechanisms underlying age-related differences in risk preference and related constructs.

Decision making is inherent to human behavior and actively shapes every aspect of our lives, from social interactions and financial management to health choices, recreation, and professional endeavors. Understanding the factors that influence decision making is thus crucial for individual and societal success. Risk preference, characterized by the tendency to pursue potentially rewarding activities despite the possibility of harm or loss (Nigg, 2017), plays an important role in decision making, and by extension, affects various primary outcomes, including individuals’ health, wealth, safety, and criminality (Epper et al., 2022; Moffitt et al., 2011). Notably, empirical work suggests age-related differences in risk preference (Frey et al., 2021; Mamerow et al., 2016; Mata et al., 2011; Rolison et al., 2013) which may affect high-stakes decisions that arise later in life, for example decisions concerning  medical treatment options or retirement savings. However, the precise nature, extent, and underlying mechanisms of age effects on risk preference remain the subject of ongoing debate.

Empirical work also suggests that risk preference is a multidimensional construct with ties (albeit of variable strength) to impulsivity and self-control (Eisenberg et al., 2019; Elliott et al., 2023; Enkavi et al., 2019; Karlsson Linnér et al., 2021; Nigg, 2017). Being closely related constructs, self-control has been taken to refer to an individual’s tendency to take deliberate actions toward a long-term objective in the face of goal-incongruent short-term interests, whereas impulsivity refers to an individual’s tendency to pursue immediate objectives that are potentially at the expense of long-term objectives (Duckworth & Steinberg, 2015). Importantly, like risk preference, impulsivity and self-control exhibit similar features of the decision context (i.e., the potential for uncertain rewards and losses), and influence decision making (Congdon & Canli, 2005; de Ridder, et al., 2018; Morales-Vives & Vigil-Colet, 2012), yet the precise theoretical status of these three constructs, their interrelationships, as well as their respective life span patterns are still unclear.

## Theoretical Accounts of Age Effects on Risk Preference, Impulsivity, and Self-Control

Several theoretical accounts of age-related differences in risk preference, impulsivity, and self-control have been proposed (for an overview of theories, see Bagaïni, Liu, Bajrami, et al., 2023; Frey et al., 2021; Seaman et al., 2022). Notably, different theories stipulate different age effects (Supplementary Table 1), including increases (Campbell et al., 2020; Carstensen, 2021; Mata et al., 2011; Shao & Lee, 2014) as well as decreases (Burr et al., 2021; Carstensen, 2021; Duckworth & Steinberg, 2015; Düzel et al., 2010; Mather et al., 2012; Mishra et al., 2016; Steinberg et al., 2008) in risk preference and related constructs across the adult human life span. Crucially, although different theories align with distinct mechanisms as primary drivers of adult age-related differences (Supplementary Table 1), some theories emphasize interrelated mechanisms that operate synergistically across various levels of analysis. For example, cognitive accounts implicate age-related changes in reward and loss sensitivity as well as in the integration of choice-relevant features and control processes (Mata et al., 2011; Olschewski et al., 2018), which have neurobiological substrates in several anatomical and functional pathways, including anticipated reward-related activity in nucleus accumbens, anticipated loss-related activity in anterior insula, as well as integrative processes subserved by activation in prefrontal cortices (Samanez-Larkin & Knutson, 2015). Moreover, cognitive and neurobiological perspectives are noteworthy for explicitly considering the impact of the measures used to capture risk preference and associated constructs (Olschewski et al., 2018; Samanez-Larkin & Knutson, 2015), and for striving to elucidate empirically observed patterns that indicate significant variability in age differences depending on the measurement employed.

## Effects of Measurement on Age Differences in Risk Preference, Impulsivity, and Self-Control

Aging studies have cumulated in the crucial insight that age differences in risk preference and related constructs vary as a function of measure. For self-report measures, observational studies based on cross-sectional (Frey et al., 2021; Mamerow et al., 2016; Rolison et al., 2013) and longitudinal data (Mata et al., 2018; Y. Liu et al., 2022) suggest decreasing risk preference across the (adult) human life span (albeit also hinting at domain-specific trajectories). In contrast, meta-analyses of studies using behavioral measures of risk preference point towards average age effects around zero, but also find evidence for variation as a function of task complexity, decision frame and domain, and maturational status (Bagaïni et al., 2023; Best & Charness, 2015; Defoe et al., 2015; Mata et al., 2011). Measurement dependence has also been reported for related constructs, including negative associations for self-report measures of impulsivity (M. Liu et al., 2020; Steinberg et al., 2008) and self-control (Sigmundsson, 2021), as well as increased self-reported future orientation in young adults compared to older children and adolescents (Steinberg et al., 2009). In contrast, for a commonly used behavioral measure, delay discounting, researchers have reported no average age effects (Seaman et al., 2022), as well as U-shaped associations with age for longer delays but no effects for shorter delays (Richter & Mata, 2018).

Cognitive accounts (Olschewski et al., 2018) point toward different modalities’ idiosyncratic decision environments as a possible moderator; behavioral measures recruit a range of cognitive processes (e.g., learning) that are susceptible to age effects, yet some of these processes may be reduced or eliminated for self-reports (Frey et al., 2021). Consequently, the judgment-formation processes underlying the rendering of self-reports may be less mutable (Steiner et al., 2021). In addition, behavioral measures have often suffered from low between-subject variability (Hedge et al., 2017); although this characteristic makes them suitable for eliciting robust group effects, this also leads to lower test–retest reliability, thus complicating their use in individual differences analyses.

## The Current Study

Amidst calls for conceptual clarity, theory development, and the refinement of measures in psychological science (Eronen & Bringmann, 2021), research in the field of aging *must* follow suit. Existing research has often focused either on age effects (and thus included a life span sample but few assessment methods) or on measurement convergence (and thus included a range of assessment methods but no life span sample). Furthermore, prior research has not consistently examined multiple constructs within the same sample, and the utilization of biological measures, including hormones and neuroimaging measures, has been infrequent. Indeed, despite age affecting the endocrine system (Piazza et al., 2010), and meta-analytic research linking testosterone with risk preference (Kurath & Mata, 2018), very few studies have controlled for hormone levels. In this study, we combined comprehensive within-subject testing of related constructs (risk preference, impulsivity, and self-control) and assessment methods (i.e., self-report, informant report, behavioral paradigms, hormone sampling, and neuroimaging) in an adult cross-sectional life span sample, with the aims of mapping points of convergence and helping to provide more comprehensive insights into questions pertaining to the constructs’ interrelations, age-related changes, and their potential mechanisms.

Our two main research questions were: First, do we find age effects on risk preference, impulsivity, and self-control, and do these converge given these constructs’ conceptual relatedness? Second, to what extent do age effects on risk preference, impulsivity, and self-control converge as a function of the assessment method? Based on extant work—including our own research (Bagaïni et al., 2023; Frey et al., 2017, 2021; Mamerow et al., 2016; Mata et al., 2011; Seaman et al., 2022; Tisdall & Mata, 2023; Tisdall et al., 2020; Y. Liu et al., 2022) and the research conducted by others (Best & Charness, 2015; Defoe et al., 2015; Eisenberg et al., 2019; Enkavi et al., 2019; Sharma et al., 2013; Strickland & Johnson, 2020)—we expected convergence (of age-related differences) for self-report measures (regardless of construct) and divergence for behavioral measures. We were more agnostic concerning the convergence of age differences in informant-report and neuroimaging measures, as these have been less systematically examined in extant work. Addressing these questions concurrently will aid the identification of *phenomena* (i.e., observations) requiring adequate description, explanation, and harmonization within a theoretical framework. Examination of functional neuroimaging indices may also help tackle mechanistic questions, such as the modality and construct specificity of reward-related processes.

## Method

All research methods were carried out in accordance with the relevant guidelines and regulations. This study was reviewed and approved by the Ethikkommission Nordwest- und Zentralschweiz EKNZ (EKNZ BASEC 2015-00094). We have previously detailed the sample, measures, and data collection in a data paper (Tisdall et al., 2024).

### Participants and Study Protocol

**Figure 1.**
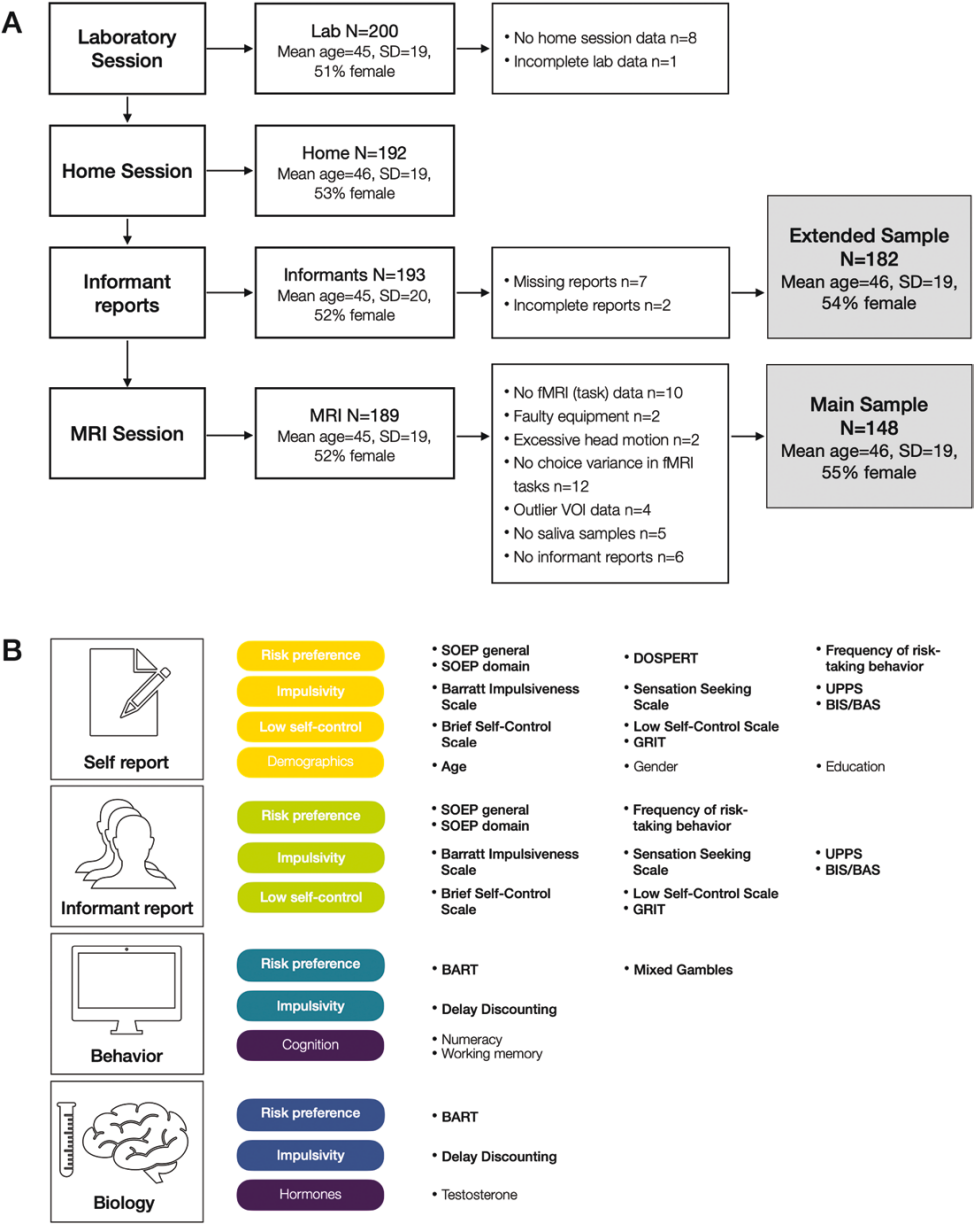
Schematic of the study protocol and measures. (A) Flow diagram of study sessions, data collection, exclusion criteria, and final samples used for analysis. Reasons for exclusion were unique to the specific sample (e.g., 18 unique exclusions to go from 200 participants in the lab session to 182 participants in the extended sample, and 41 unique exclusions to go from 189 participants in the MRI session to 148 participants in the main sample), thus do not necessarily add up between the two samples. (B) Overview of measures collected as part of the within-participant study design. We use the same colors to organize and distinguish the results for different measures. Measures in bold were used as predictor or outcome variables, measures in regular font were used as covariates. See Supplementary Table 3 for further details.

Informed by power analyses (Supplementary Material, p. 3), we recruited a convenience sample of 200 healthy, right-handed volunteers between 16 and 81 years of age with no history of neurological disorders, who met all MRI safety requirements, and who had normal or corrected to normal vision (Supplementary Material, p. 3). Participants completed three study sessions (Figure 1A): an in-person laboratory session, a home session, and a neuroimaging session approximately 1–2 weeks after the laboratory session. A schematic overview of the study components and their characteristics is presented in Supplementary Table 2. All sessions were incentivized; participants received a financial reimbursement of 15 Swiss francs per hour of participation (approximately 98 Swiss francs, ~98 U.S. dollars, for participation in all three study sessions), and could further earn a financial bonus based on their performance in the behavioral measures. In addition to collecting data from the participants, we collected data about the participants from up to three participant-nominated informants (Figure 1A, Supplementary Material, pp. 5–6). As reimbursement for their time, informants received a voucher for 10 Swiss francs (~10 U.S. dollars) for local shops and attractions.

### Data Collection and Individual Measures

We collected a large battery of measures (Figure 1B, Supplementary Table 3), which we selected based on their (a) common use to capture risk preference, impulsivity, and self-control, and (b) implementation under different modalities (i.e., self-report, informant report, behavior, and neuroimaging). Where feasible, we collected multiple measures to capture the same construct, and planned to perform dimensionality reduction to extract more robust indices for the main analyses. We also assessed demographic characteristics and cognitive capacity and used these as covariates (Frey et al., 2021). To limit participant burden, informants completed a subset of the self-report items completed by the participants (Figure 1B, Supplementary Table 3, Supplementary Material, p. 6).

### Data Preprocessing

A detailed description of all preprocessing steps (e.g., dimensionality reduction, preprocessing of neuroimaging data), as well as any deviations from the analysis plan and preregistration, are documented in the Supplementary Material (pp. 15–32). We briefly outline the main components of the data preprocessing pipeline below.

#### Independent-analysts approach

The analysis of data originating from the behavioral measures (Supplementary Figures 1 and 2) was conducted independently by analysts with specific expertise in the particular measures (authors R. Frey, D.U. Wulff, D. Kellen, R. Mata, L. Tisdall). Analysts were unconstrained in their approach to data analysis and independently formulated an analysis protocol for their particular measure(s) prior to receiving the data. R. Frey analyzed data from the Balloon Analogue Risk Task (BART), D.U. Wulff analyzed data from the delay discounting task, and D. Kellen analyzed data from the mixed gambles task. R. Mata analyzed the extracted testosterone data, and L. Tisdall analyzed self-report and informant-report measures, data from the functional magnetic resonance imaging session, and covariates.

#### Study completions, missing data, and samples used for analysis

To perform analyses on complete data sets while retaining as many participants as possible, we created two inclusive samples (Figure 1A). The main sample comprised 148 participants (mean age = 45.50 years, standard deviation [*SD*] age = 19.24 years, age range = 16.15–81.38 years) for which we obtained data from all sessions and measures, including biomarker data from the MRI session and saliva samples. The main sample comprised 82 female participants (55.41%, mean age = 43.16 years, *SD* age = 18.37 years, age range = 16.15–80.63 years) and 66 male participants (44.59%, mean age = 48.40 years, *SD* age = 20.03 years, age range = 17.37–81.38 years). The extended sample comprised a wider pool of 182 participants (*n* female = 98, mean age = 45.96 years, *SD* age = 19.44 years, age range = 16.15–81.38 years); for these participants, we collected all but the biomarker data. The two nested samples were comparable with regard to their respective (socio)demographic characteristics (Supplementary Figure 3).

#### Dimensionality reduction of self-report and informant-report measures

As laid out in our preregistration, we intended to perform confirmatory factor analysis using a bifactor model to achieve dimensionality reduction of self-report indices. To achieve coherent outcome variables, we coded all measures such that higher scores express a higher preference for or frequency of risky options, higher impulsivity, and lower self-control. This transformation also means we would expect positive correlations between the constructs (e.g., between impulsivity and low self-control). However, fit indices for the bifactor model were poor (Supplementary Material, pp. 17–18). Consequently, we deviated from our preregistration and adopted an alternative dimensionality reduction approach that minimized the risk of overfitting. Specifically, we extracted unit-weighted construct-specific and construct-independent (general) sum scores for both self-report and informant-report data (Supplementary Figures 4 and 5). In brief, we first normalized participants’ scores within (sub)scales, and then added the normalized scores for different sets of scales, each contributing equally (i.e., with a weight of one) to the overall score. For details, see the Supplementary Material (pp. 18–20).

#### Performance-based indices from behavioral measures

Data from the behavioral measures collected during the lab session were preprocessed and performance-based indices were extracted by individual analysts (Supplementary Material, pp. 20–24). Following planned analyses, we computed the adjusted number of pumps as an indicator of risk preference in the BART (Supplementary Figure 6), extracted the proportion of smaller–sooner choices from the delay discounting data, and computed the proportion of risky choices from the mixed gambles data (Supplementary Figure 7).

#### Hormone markers

Participants’ saliva samples were externally assayed for testosterone. For the individual differences analyses, we produced one index by averaging testosterone levels across all available measurements per participant (Supplementary Material, pp. 24–25).

#### Neuroimaging data

As a general overview, first we preprocessed the fMRI data from the two measures completed inside the scanner (BART, delay discounting) following standard preprocessing pipelines using SPM12 (Supplementary Material, pp. 25–26). Second, we generated input models to estimate brain activation differences for predefined contrast analyses for the BART (*pumps on reward balloons > pumps on control balloons*) and delay discounting (*smaller–sooner rewards > larger–later rewards*), and checked group-level activation maps for activation patterns commonly reported in the literature (Supplementary Figure 8; Supplementary Material, pp. 26–29). Third, we generated output models via the extraction of brain activation differences from the contrast analyses for three predetermined volumes of interest: the medial prefrontal cortex, the anterior insula, and the nucleus accumbens (Supplementary Figure 9; Supplementary Material, pp. 29–31).

#### Variable set used for analysis

The result of the preprocessing pipeline was a set of variables that we used for all key analyses of the main sample (Table 1). All analyses included age, general, and construct-specific self-report as well as informant-report composite indices, three indices from the behavioral measures, and four indices of brain activation differences in the BART and delay discounting. Given their potential role as moderators, we further included five covariates in all analyses: gender, education, numeracy, working memory (examined using the automated operation span task), and testosterone (Frey et al., 2021; Kurath & Mata, 2018).

**Table 1.** Variable Set Used in All Analyses of the Main Sample (*N* = 148)

Variable	Label	Description
Predictor	age	Age in years
Outcomes	SR	Unit-weighted self-report score
	SRrisk	Unit-weighted risk preference score (self-reports)
	SRimp	Unit-weighted impulsivity score (self-reports)
	SRlsc	Unit-weighted low self-control score (self-reports)
	IR	Unit-weighted informant-report score
	IRrisk	Unit-weighted risk preference score (informant reports)
	IRimp	Unit-weighted impulsivity score (informant reports)
	IRlsc	Unit-weighted low self-control score (informant reports)
	BEHbart	Adjusted number of pumps (BART)
	BEHla	Proportion of risky choices (mixed gambles)
	BEHdd	Proportion of smaller–sooner choices (delay discounting)
	BIObart_nacc	Mean NAcc beta Pumps_Rew_ > Pumps_Con_ (BART)
	BIObart_ains	Mean AIns beta Pumps_Rew_ > Pumps_Con_ (BART)
	BIOdd_nacc	Mean NAcc beta Choices_SS_ > Choices_LL_ (delay discounting)
	BIOdd_mpfc	Mean MPFC beta Choices_SS_ > Choices_LL_ (delay discounting)
Covariates	gender	Male/female
	edu	Highest level of education
	numcy	Number of correctly solved problems (numeracy task)
	ospan	Number of correctly recalled letters (operation span task)
	tstrn	Mean testosterone (picograms/milliliter)

*Notes*: AIns = anterior insular cortex; BART = Balloon Analogue Risk Task; BEH = behavior; BIO = biological measure; Choice_SS_ = smaller–sooner choices; Choice_LL_ = larger–later choices; IR = informant report; MPFC = medial prefrontal cortex; NAcc = nucleus accumbens; Pumps_Rew_ = Pumps on reward balloons; Pumps_Con_ = Pumps on control balloons; SR = self-report.

### Main Analyses

To identify which of the variables formed clusters based on their associations with all other variables, we first sought to visualize the variable space and associations between the predictor (age), outcome variables, and covariates by generating network plots. The network plots were purely exploratory in nature and did not inform the subsequent analyses.

In the second step, we adopted a multiverse analysis approach for the main analyses, and exhaustively examined associations between outcome variables, predictors, and all possible, nonredundant combinations of covariates in a comprehensive yet (visually) accessible way via specification curve analysis (SCA; Simonsohn et al., 2020). The SCA for the main sample included 1 predictor (age), 5 covariates (gender, education, numeracy, working memory, testosterone), and 15 outcome variables (4 indices from the analysis of self-reports, 4 indices from the analysis of informant reports, 3 indices stemming from the behavioral measures, and 4 indices capturing brain function in the fMRI versions of the BART and delay discounting). This additive combinatorial approach resulted in 15 × 2^5^ = 480 unique specifications for the main sample, which we estimated using ordinary least squares regression models, implemented in the R package *specr* (Masur & Scharkow, 2020). To ascertain the global significance of the observed specification curves, in a third step, we followed a permutation-based approach (Rohrer et al., 2017) to estimate whether the empirical specification curves deviated systematically from the to-be-expected false-positive effects if there was, in fact, no systematic relationship between the predictor and outcomes (i.e., the null distribution of effect sizes). All main analyses were conducted in R Studio (R Core Team, 2021), unless otherwise specified.

## Results

We report results from the main sample here and provide additional results for the extended sample in the Supplementary Material (pp. 33–42). Overall, the results obtained for the extended sample mirrored those of the main sample. Following the variable transformation applied to self-control scales, we report results in relation to low self-control.

### Bivariate Associations

**Figure 2.**
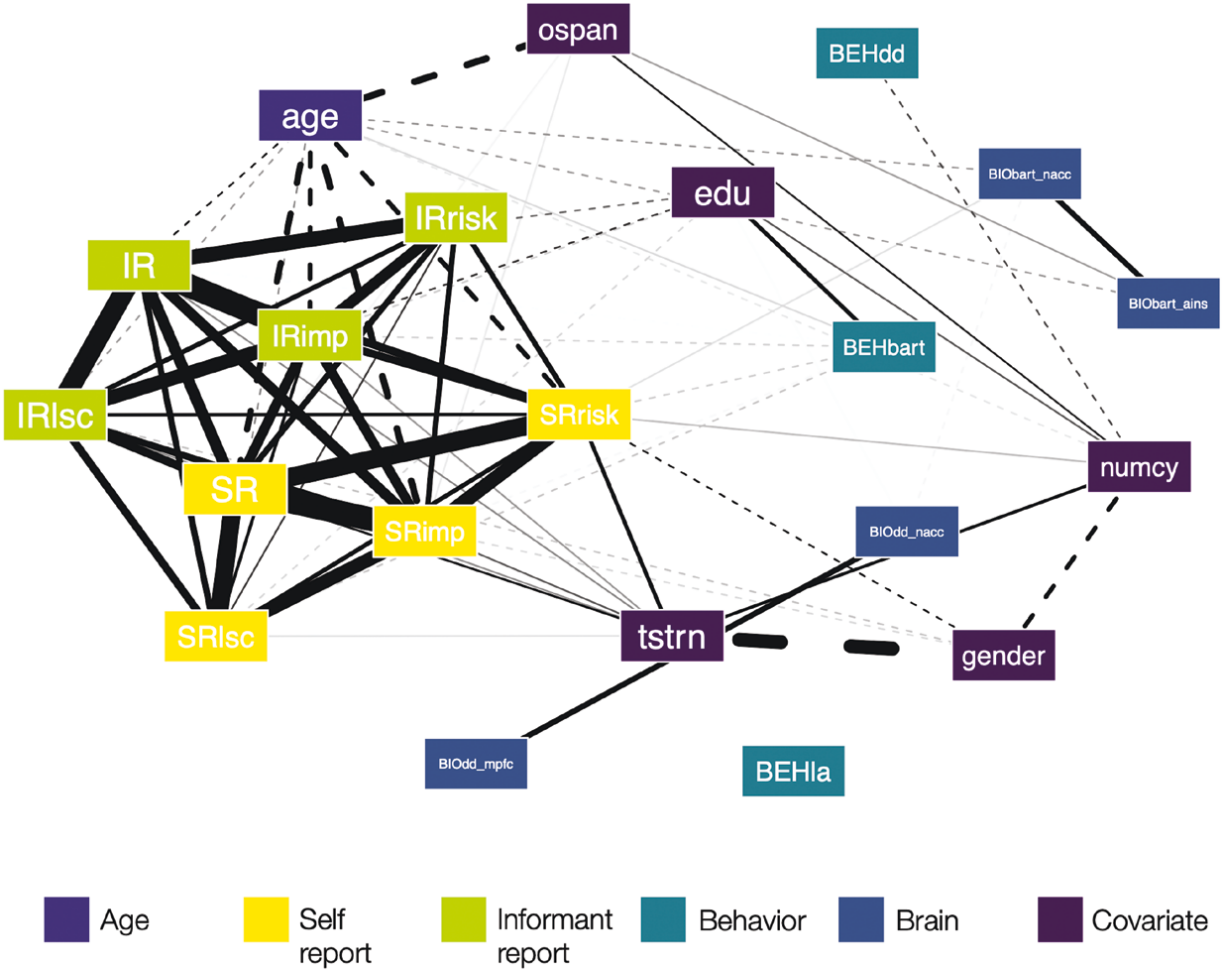
Network plot of bivariate correlation coefficients between study variables (main sample, *N* = 148). Variables were visually grouped by measurement modality. Edge thickness is indicative of the strength of Pearson’s correlation coefficient between two variables, with solid (dotted) edges representing positive (negative) associations. We only plotted correlations with absolute coefficients of *r* ≥ 0.15. For labels, see Table 1.

Based on bivariate correlations between all variables entered into the main analyses (Supplementary Figure 10), we generated network plots to visualize the variable space and associations between the predictor (age), outcome variables, and covariates in the main sample (Figure 2; Supplementary Figure 11 for the extended sample network plot). We visually grouped variables by measure to illustrate the (lack of) convergence between different assessment methods as well as their association with age and covariates.

Focusing on age effects, convergent negative associations emerged for age and risk preference, impulsivity, and low self-control, but only for self- and informant reports. In contrast, age was not associated with behavioral indicators of risk preference or related constructs, and was inconsistently associated with neural indices. As expected, age was negatively associated with indices of cognitive capacity (i.e., working memory and numeracy).

Focusing on measurement modality, we observed (a) strong convergence (i.e., positive correlations) for self- and informant report, regardless of construct, (b) low convergence within the group of behavioral measures as well as between behavioral and self-report measures, (c) strong, construct-independent convergence between self-report and informant report, and (d) strong convergence between neural indices originating from the same fMRI measure, but not between neural indices and self, informant, or behavioral indices.

In line with previous work (Kurath & Mata, 2018), testosterone was consistently positively associated with self- and informant-report indices of risk preference, impulsivity, and low self-control (mean *r*_Pearson_ = 0.22, *SD* = 0.08), but not or weakly negatively with behavioral indices from the BART, delay discounting, or mixed gambles.

In summary, bivariate associations provided the first set of results suggestive of convergent age effects on risk preference, impulsivity, and low self-control, but also indicated that age effects are dependent on the assessment method used. To estimate the robustness of these associations, in particular with regard to the role of different (cognitive) covariates and operationalizations for the observed age effects, we adopted a multiverse analysis approach.

### Multiverse Analysis: Specification Curves

**Figure 3.**
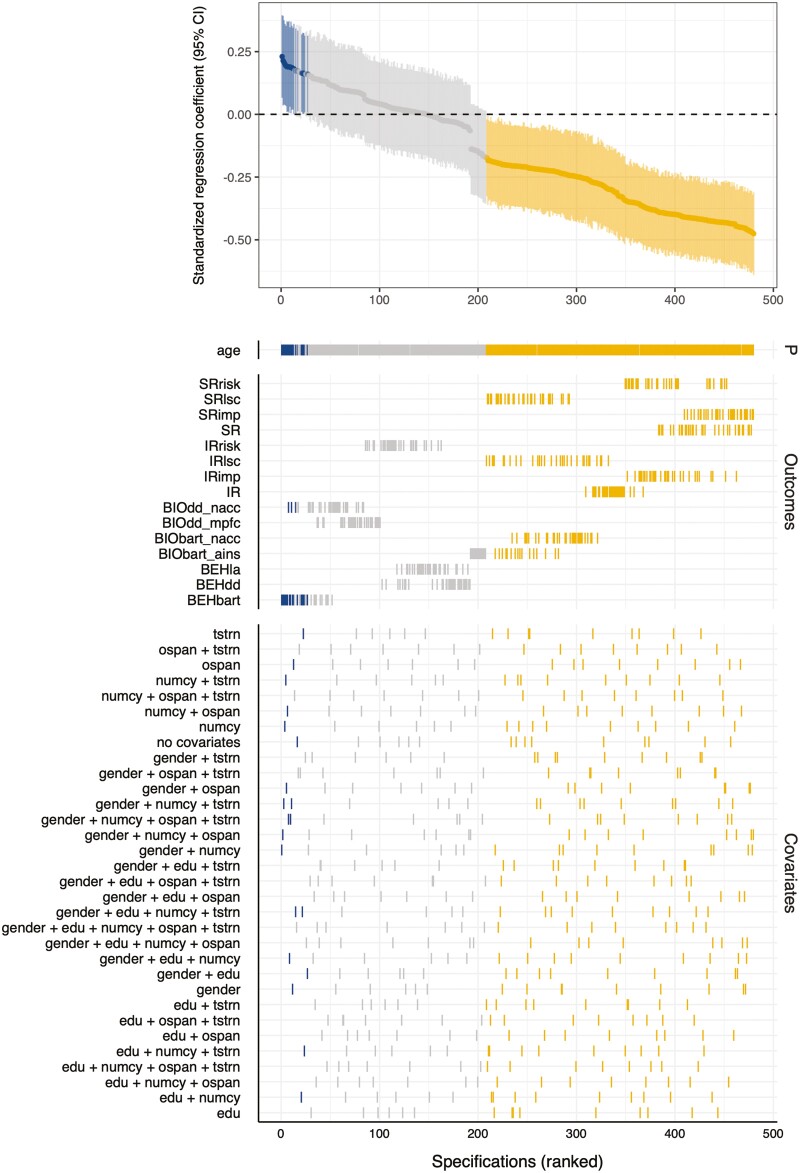
Results of the SCA of age effects on risk preference and related constructs in the main sample (*N* = 148). The top graph displays the standardized regression coefficients (95% CI) for age on varying operationalizations of risk preference, impulsivity, and low self-control. The specifications were ordered by effect size, from positive to negative. Tick marks in the lower panels describe the exact specifications, including which outcome and which covariate(s) were included in a given specification. P = predictor. Colors indicate significant (*p* ≤ .05) positive (blue) and negative (orange) effects. For labels, see Table 1.

To comprehensively assess and visualize the association between age and varying indicators of risk, impulsivity, and low self-control while controlling for a range of potential covariates, we performed SCA. For the main sample, we estimated 480 specifications, 188 (39.2%) of which returned null effects (i.e., beta coefficients for age that were not significantly different from zero at *p* ≤ .05), 20 (4.2%) positive effects, and 272 (56.7%) negative effects (Supplementary Table 4; comparable results were observed for the extended sample, Supplementary Table 5). Aggregating across all 480 specifications, the median effect of age for the main sample was −0.21. As foreshadowed by the bivariate associations (Figure 2), the result of predominantly negative effects of age on risk preference and related constructs from self- and informant reports is also easily discernible from the accompanying specification curve plots for the main sample (Figure 3; Supplementary Figure 12 for the extended sample SCA).

Concerning self- and informant-report measures, age was negatively associated with indices of self-reported risk preference (SRrisk), impulsivity (SRimp), and low self-control (SRlsc), as well as a general self-report index (SR), controlling exhaustively for (linear, additive combinations of) gender, education, numeracy, working memory, and testosterone. Age effects were strongest (as indicated by the absolute magnitude of the standardized beta coefficients) for impulsivity, followed by risk and low self-control. We found similar effects for informant reports for impulsivity (IRimp) and low self-control (IRlsc), as well as the domain-general indicator of informant report (IR). Age effects for informant reports were also strongest for impulsivity.

Concerning behavioral measures, specifications that estimated the effect of age on indicators derived from mixed gambles and a delay discounting task yielded coefficients around zero, regardless of the covariate(s) included in the different specifications. In contrast, we obtained a set of positive effects of age on risk taking in the BART, but these were dependent on the specific (set of) covariates included in a given specification; approximately half of all BART models indicated positive age effects, and half indicated age effects around zero, depending on which covariates were included in the model.

Concerning neuroimaging measures, we observed negative effects of age on activation differences in nucleus accumbens and anterior insula for the BART. Regarding the former, our results suggest that as individuals grow older, they may experience blunted activity in the nucleus accumbens for anticipated (but uncertain) rewards, yet previous studies have reported preserved nucleus accumbens activation during reward anticipation (Samanez-Larkin & Knutson, 2015). The fact that the BART involves learning, which, in turn, may affect the reward signal, could account for the seemingly conflicting findings. Concerning the latter, our anterior insula findings are consistent with neurobiological predictions for age effects on neural correlates of (risky) decision making (Samanez-Larkin & Knutson, 2015); relative to younger adults, older adults showed decreased activation in brain regions associated with loss anticipation, specifically the anterior insula. Confirming the crucial role of behavioral measures in brain–behavior associations (Tisdall et al., 2020), and by extension age effects, we obtained mainly insignificant specifications for neural markers extracted from delay discounting. We also obtained a small set (*n* = 3) of significant specifications indicating positive age effects on nucleus accumbens activation differences for smaller–sooner versus larger–later choices, but these were dependent on the covariate(s) included in the model, and as such less credible than the other age effects revealed by our analyses. Across constructs and assessment methods, the inclusion of testosterone as a biological covariate in the separate specifications does not seem to have systematically affected the overall pattern of age effects.

In summary, multiverse analyses confirmed our initial analyses using bivariate correlations, indicating that age effects on risk preference and related constructs converge, but also that these are modality-dependent. The adoption of a multiverse approach allowed us to identify an interesting heterogeneity of behavioral effects for the BART, which may speak to theoretical accounts invoking measurement-specific characteristics such as working memory as potential confounds for age effects (Mata et al., 2011; Olschewski et al., 2018). Furthermore, although age effects were evident for *both* reward- and loss-related brain activation, this finding only extended to one behavioral measure (BART).

### Permutation Testing

We assessed the probability of the set of observed age effects being false-positive results via the implementation of permutation tests. When considering all specifications jointly, these analyses corroborated the robustness of our empirical SCA results: Based on 500 data sets for which we randomly sampled the age variable with replacement, none resulted in as many as or more than 292 (i.e., the number of significant specifications observed in the original data set) significant specifications. In other words, the probability of observing the number of effects that we obtained from our original SCA under the null hypothesis was zero (Supplementary Table 4, Supplementary Figure 13), speaking to the robustness of our results. A similar pattern was observed for the extended sample (Supplementary Table 5, Supplementary Figure 14).

### Exploratory Analyses of Convergence Between Self- and Informant-Report Indices

Our analyses suggested considerable convergence between self- and informant-report measures, but do not speak to putative mechanisms. To help address this issue, we performed exploratory analyses to further understand whether informant reports capture idiosyncratic information about the target person (i.e., study participant) or converge with self-report indices due to other factors (e.g., aging stereotypes). Correlations obtained for counterfactual pairings were consistently lower than for bootstrapped true participant–informant pairings (Supplementary Figure 15), and regression analyses suggested that informants used information beyond demographics about the to-be-judged person for their ratings (Supplementary Tables 6 and 7). Although further work on the underlying mechanisms is required, these initial findings suggest that informant reports may provide a useful elicitation method to capture individual differences in risk preference, impulsivity, and low self-control.

## Discussion

The starting point for examining age differences in risk preference, impulsivity, and self-control was the recognition that a holistic assessment of the constructs, measures, and potential mechanisms is currently lacking. In this study, we aimed to map the landscape of age differences and thereby bring to the fore the *phenomena* in need of adequate description, explanation, and harmonization (Eronen & Bringmann, 2021).

The results of our multiverse analysis approach suggest converging negative effects of age on risk preference, impulsivity, and low self-control across the adult human life span (Frey et al., 2021; M. Liu et al., 2020; Seaman et al., 2022; Sigmundsson, 2021; Steinberg et al., 2008; Y. Liu et al., 2022), and reinforce the conclusion that age effects are not independent of the assessment method (Best & Charness, 2015; Mata et al., 2011; Seaman et al., 2022; Sharma et al., 2013; Strickland & Johnson, 2020): Homogeneous negative effects of age were obtained for indices captured via self- and informant reports, but behavioral and neural indicators were not consistently associated with age. Regarding mechanisms, the negative effects of age on neural activation differences in the anterior insula for risk-related decisions in the BART are compatible with an age-related positivity bias (Carstensen, 2021), whereby older adults focus less on the potential loss or evaluate the potential loss as less negative. However, we cannot unequivocally attribute the observed age effect on behavior to loss-related mechanisms due to a lack of associations between neural markers and behavioral measures. Furthermore, negative age effects on nucleus accumbens activation differences have been reported for paradigms with probabilistic learning components (such as the BART), whereby older adults may experience difficulty in the learning and appropriate updating of option values from probabilistic feedback (Samanez-Larkin & Knutson, 2015). In our analyses, many of the significant specifications showing a positive effect of age on BART behavior did not control for working memory, whereas most of the insignificant specifications included working memory as a covariate. Yet, the lack of associations between nucleus accumbens activation and BART behavior suggests that this reward-related mechanism in isolation is unlikely to be driving age effects. Supporting previous neuroimaging research (Seaman et al., 2018), we found no (credible) age effects on neural markers in a delay discounting paradigm, suggesting preserved temporal discounting across the adult human life span.

Three limitations concerning the behavioral measures in this study require mentioning. First, we chose relatively short delay periods that were in line with past work and manageable to implement in our study involving real payoffs (Eppinger et al., 2012; van den Bos et al., 2014). However, in light of recent research pointing toward the dependence of age effects on long(er) delay intervals (Leverett et al., 2022; Richter & Mata, 2018), this choice can be seen as a limitation. Second, age differences have been observed for choices between risky and certain options (Mather et al., 2012), whereas we manipulated risk through the sign and magnitude of outcomes rather than probabilities. Our choice of gambles was intended to optimize the estimation of participants’ loss-aversion parameter, yet due to the high correlation between the loss-aversion parameter lambda and the proportion of risky choices (*r*_Spearman_ = −0.86), we opted to run our analyses on the performance index rather than on the model-based estimate. To understand the extent to which age differences vary as a function of the types of options presented in description-based risky decision making requires further examination and research synthesis. Third, only the behavioral but not the self- or informant-report measures were incentivized, which could be a contributing factor to the (lack of) age-related differences (Seaman et al., 2016). Although meta-analytic work suggests that whether incentives are merely hypothetical or are realized does not affect age effects on risk or uncertainty-related decision making (Bagaïni et al., 2023; Seaman et al., 2022), exploring methods to either incentivize self-report measures or detach behavioral measures from (hypothetical) incentive structures to ensure comparable indices is an intriguing question meriting investigation.

Looking ahead, we concur with Eronen and Bringmann’s (2021) assessment that *conceptual clarification* and *phenomena detection* will be crucial requisites for psychological science research. Regarding the former, our analyses revealed a substantial overlap between risk preference, impulsivity, and low self-control, and their respective age differences, reinforcing the notion that risk preference is a multidimensional construct linked to impulsivity and low self-control (Eisenberg et al., 2019; Elliott et al., 2023; Nigg, 2017). To establish a firm understanding of these related constructs and decompose their variance into shared and unique components, previous studies have started to address the question of the psychometric structure of risk preference and related constructs (Eisenberg et al., 2019; Frey et al., 2017), yet they do not fully speak to the generalizability and stability of the extracted factors across the human life span. Unfortunately, our planned psychometric analyses resulted in unsatisfactory model fits, which could be due to a number of methodological and conceptual reasons. Methodologically, we analyzed a smaller number of measures and, more importantly, a substantially smaller sample compared to the original analyses conducted to identify the psychometric structure of risk preference (Frey et al., 2017) using a confirmatory approach. Conceptually, previously proposed psychometric solutions may present psychometric snapshots of young adult samples. A central goal for future work should, therefore, include the assessment of configural, metric, and scalar invariance of proposed psychometric model structures, which may require the comprehensive identification, harvesting, (meta)analysis, and extension of existing longitudinal efforts (Moffitt et al., 2011). The achievement of conceptual clarification will allow researchers to adopt measures in a more principled, construct-specific way (Eisenberg et al., 2019; Enkavi et al., 2019; Sharma et al., 2013; Strickland & Johnson, 2020).

This brings us to our second proposal for how to move forward, namely to engage in earnest with *phenomena detection* (Eronen & Bringmann, 2021) and curate better, more extensive sets of observations. In the context of research on (age differences in) risk preferences, we see three main routes for how improved data collection can form the basis for the refinement of extant and formulation of new theories: multimodal and multiconstruct data, development of behavioral measures, and triangulation with objective indices.

First, as we have done here, collecting data on the same construct using different modalities can guide our search for mechanisms. For example, although the convergence between self- and informant reports of risk preference, impulsivity, and low self-control clearly speaks to the complementary nature of these two elicitation methods (McAbee & Connelly, 2016), it also suggests that convergence might be traced back to similar cognitive processes, for example, in rendering self- and informant reports. The cognitive processes subserving self-reported risk preference (e.g., evidence integration from memory) appear to be temporally stable and predictive of self-report scores (Arslan et al., 2020; Steiner et al., 2021), but whether these findings apply equally to related constructs, informant reports, and, most importantly, are characterized by age effects, remains to be tested. Our exploratory analyses suggest that informants are using idiosyncratic information beyond demographics about the to-be-judged individual, but a more comprehensive research program is required to pinpoint the cognitive, affective, and motivational processes that are fundamental to attitudinal measures, and compare these to the processes thought to subserve behavioral measures.

Second, we adopted commonly used behavioral measures to capture individual differences in risk preference, impulsivity, and low self-control. However, it has been pointed out that many behavioral measures (and their neuroimaging counterparts) are confounded, thus challenging attempts to isolate the exact mechanisms of interest, particularly in individual differences research (Olschewski et al., 2018). Furthermore, the preferred adoption of certain behavioral measures for certain constructs may lead to confounding measures and constructs (Nigg, 2017). To further progress, we will need to engage with the challenging task of measurement development. Crucially, measurement development needs to consider the purpose; that is, whether behavioral measures should capture intraindividual, interindividual, or group differences (Hedge et al., 2017). As a result of such development and innovation, we may yet find converging age effects in risk preference, impulsivity, and low self-control captured by behavioral measures, which may bring us closer to disentangling the mechanistic role of decision-relevant features such as rewards, losses, or uncertainty.

Third, our results only speak to the convergence of different measures, but not their accuracy (i.e., predictive validity). To quantify the alignment between convergence and accuracy, researchers need to develop efficient, ethical procedures to collect “real-life” objective indicators of the constructs, attitudes, and behaviors under investigation (Epper et al., 2022; Moffitt et al., 2011), for example, in the domain of health (e.g., visits to the emergency department), wealth (e.g., investment portfolios), and delinquency (e.g., arrest records). These objective measures can then be used to triangulate empirical findings from other modalities, uncover convergence gradients between modalities, and, importantly, establish a ground truth that we aim to describe, explain, and predict.

At a time when psychological science is being challenged by calls for conceptual clarification, development of theories, and the fine-tuning of measures (Eronen & Bringmann, 2021), psychological research in the field of aging *can* follow suit. Although we did not directly test motivational or affective accounts, our results overall support the proposed negative effects of age on risk preference and related constructs. Moreover, our results align with both cognitive and neurobiological theories stipulating age effects on risk preference via reward- and loss-related processes, and their proposed neural substrates. Most importantly, the observed convergent and divergent data patterns are the *phenomena* in need of harmonization and explanation; they can help to pinpoint blind spots in existing theories and lay the groundwork for constructing new theories that address these shortcomings.

## Supplementary Material

gbae092_suppl_Supplementary_Materials

## Data Availability

We use data from the AgeRisk study, for which we have made all data publicly available via the Open Science Framework (https://osf.io/sh34n/), and OpenNeuro (https://openneuro.org/datasets/ds004711), and which we have documented in detail in a data paper (Tisdall et al., 2024). The analyses presented here were preregistered via aspredicted.org (https://aspredicted.org/98R_QYR). The analysis plan, data, and code supporting the main analyses reported in this paper are available on the Open Science Framework (https://osf.io/46uys/). A preprint of this paper has been uploaded to OSF Preprints (https://osf.io/preprints/osf/uj359). We have previously reported additional in-depth, exploratory contrast analyses of the neuroimaging data (Tisdall & Mata, 2023), which were not part of this preregistration.
