# Supplementary material for: Convergence of Age Differences in Risk Preference, Impulsivity, and Self-Control: A Multiverse Analysis
Source: J Gerontol B Psychol Sci Soc Sci. 2024 May 23;79(8):gbae092. doi: 10.1093/geronb/gbae092 (PMC11237994; doi:10.1093/geronb/gbae092)
Supplement: gbae092_suppl_Supplementary_Materials [file gbae092_suppl_supplementary_materials.pdf]

The Journals of Gerontology, Series B: Psychological Sciences and Social Sciences

Supplementary Material: Loreen Tisdall, Renato Frey, Dirk U. Wulff, David Kellen, & Rui Mata. Convergence of age differences in risk preference, impulsivity, and self-control: A multiverse analysis

SUPPLEMENTARY MATERIAL

SUPPLEMENTARY INFORMATION

Supplementary Table 1. Overview of selected mechanisms underlying effects of age on risk preference, impulsivity, and self-control.

	Direction of effect of age on:		
Mechanisms	Risk preference	Impulsivity	Self-Control
Motivations and life goals (Carstensen, 2021; Depping & Freund, 2011; Mather et al., 2012)	-	+/-	.
Functional / resource accumulation (Mishra et al., 2016)	-	.	.
Affect and mood (Burr et al., 2021; Shao & Lee, 2014)	+/-	+/-	.
Cognitive control (Duckworth & Steinberg, 2015; Steinberg et al., 2008)	-	-	+
Inhibitory deficits (cf. Campbell et al., 2020)	.	+	.
Cognitive complexity of the task (Mata et al., 2011; Olschewski et al., 2018)	+/-	+/-	.
Dopaminergic function (Düzel et al., 2010)	-	-	.
Brain structure and function (Samanez-Larkin & Knutson, 2015)	+/-	+/-	.

Note: "+" = increase as a function of age; "-" = decrease as a function of age; "." = no construct-specific account.

SUPPLEMENTARY METHODS

Power calculation

We adopted a heuristic approach to calculating power for the cross-sectional analyses in G*Power using standard methods under the assumption of using linear regression models to estimate age effects on the primary outcomes of interest. A sample larger than 80 participants was determined to yield 80% power to detect medium effect sizes in cross-sectional analyses. The comparatively larger recruited sample reflects our interest in individual differences analyses, as well as our intention at the time of study planning to extend the study to a longitudinal design (which has not been implemented); both components would require a sufficiently large (initial) sample to achieve and retain sufficient power. Moreover, for the desired longitudinal analyses, simulation methods assuming the use of bivariate latent growth curve models suggested 200 individuals to be sufficient to detect medium-size effects with 80% probability.

Exclusion criteria during the screening stage

We excluded individuals from participation based on contraindications for the magnetic resonance imaging (MRI) component of the study. In particular, we excluded individuals from participation if screening indicated any of the following to apply: permanent implants (e.g. pacemaker, cochlear implant, neurostimulators, insulin pump), claustrophobia, tinnitus, epilepsy, pregnancy, fixed metal in or on the body (e.g., surgical clips, metal splinters, metal prosthesis, copper coils, artificial heart valves), having had heart or brain surgery, any conditions which prevented individuals from lying (comfortably) still inside the scanner bore, as well as the use of any prescribed medication which could interfere with cognitive and neural function. We also excluded individuals if their vision was impaired to the extent that it could not be sufficiently corrected inside the scanner, either via participants' own contact lenses or MRI-safe glasses provided at the scanning facility.

Study components and characteristics

Supplementary Table 2 provides an overview of the study components, sessions, and their respective characteristics.

Supplementary Table 2. Study components and characteristics.

	Screening	Laboratory session	Home session	MRI session	Informants
**Informed consent**		Y	Y	Y	Y
**Data collection mode**	phone and mail	computerized and on paper, in-person lab visit	online or on paper, at home	biological, computerized	mail
**Demographic data**	Y	Y	-	Y	Y
**Self-report measures**	-	Y	Y	Y	Y
**Behavioral measures**	-	Y	-	Y (behavior on fMRI paradigms)	-
**Cognitive capacity measures**	-	Y	-	-	-
**Biological measures**	-	-	Y (saliva)	Y (brain function)	-
**Informant-reports**	-	Y (nomination of up to three informants)	-	-	Y
**Incentivized**	-	Y	Y	Y	Y
**Duration (minutes)**	20-30	180	90	120	30

Note: Y = yes, present.

Data collection and individual measures

The main aim of this study was to robustly estimate the effect of age on risk preference and related constructs (i.e., impulsivity and low self-control), focusing on whether the age effect differs by measurement modality (i.e., self-report, informant-report, behavioral measures, brain function) while controlling for covariates (gender, education, cognitive capacity, hormonal differences). For this purpose, we collected an extensive battery of measures (Supplementary Table 3). An exhaustive list of all measures collected over the three study sessions can be found in our analysis plan.

Self-reports. Self-report measures were collected during the lab, home, and neuroimaging session. During the lab and neuroimaging session, we collected all self-report measures in electronic form via surveys programmed in Unipark (https://www.unipark.com/en/). All computer-based self-reports were programmed to alert participants to missing responses to facilitate complete data records. To reduce participant burden and retain participants between sessions, participants were able to select between completing the self-reports that were the subject of the home session either online (via personalized study links) or via personalized pen-and-paper study packs. Both online and pen-and-paper questionnaires were completed prior to the neuroimaging session. Pen-and-paper packs were returned during the neuroimaging session and responses were added to the online questionnaire platform by the research team. For quality assurance of the transcription from analog to digital responses, one author (L.T.) compared randomly selected pen-and-paper responses to their respective electronic entries.

Informant-reports. Informant-reports from parents and teachers have been collected as part of various developmental and longitudinal research designs (Moffitt et al., 2011) because they provide a unique source of information (McAbee & Connelly, 2016), yet their relationship to self-report or behavioral measures of risk preference and related constructs, as well as to age, have not yet been scrutinized. To obtain informant-reports, we asked study participants after the laboratory session to nominate up to three individuals of their choice to act as informants. Upon their nomination by the participant, informants were contacted via post and informed of the study and their nomination to act as an informant for the participant. Informants were provided with written study materials, a consent form, a printed informant survey, a labeled return envelope, and a voucher worth 10 Swiss francs (~10 U.S. dollars) for local shops and amenities as reimbursement for their time. The informant surveys included items about the informant's age, gender, duration, and type of relationship with the study participant, as well

as ratings of the study participant's risk preference, impulsivity, and low self-control. To reduce the informant burden, we asked informants to rate a subset of 54 items completed by study participants. In particular, informants rated study participant's general and domain-specific (recreation/sport and trust in strangers) risk preference (TNS Infratest Sozialforschung, 2014), 12 items (two per factor) from the Barratt Impulsiveness Scale, Version 11 (Patton et al., 1995; Preuss et al., 2008), eight items (two per factor) from the UPPS Impulsive Behavior Scale (Schmidt et al., 2008; Whiteside & Lynam, 2001), eight items (two per factor, easy and difficult) from the Sensation Seeking Scale (Beauducel et al., 2003; Zuckerman et al., 1978), four items (two per factor) from the BIS/BAS Scale (Carver & White, 1994; Strobel et al., 2001), three items (highest loading item plus two tied second highest loading items) from the Brief Self-Control Scale (Bertrams & Dickhäuser, 2009; Tangney et al., 2004), 12 items (two per factor) from the Low Self-Control Scale (Grasmick et al., 1993; Seipel, 2014), and four items (two per factor) from the GRIT Scale (Duckworth et al., 2007; Fleckenstein et al., 2014).

For the main sample comprising 148 participants, we collected informant-reports from 448 informants (average of 2.5 informant-reports per participant). We received three informant-reports for 93 participants (62.8%), two informant-reports for 32 participants (21.6%), and one informant-report for 23 participants (15.5%). The majority of informants was female (229, 62.6%; one informant did not disclose their gender), informants were on average 44.6 years old (SD = 18.1, range = 16–86 years), and had an average relationship duration with the participant of 22.5 years (SD = 15.9, range = 1–73 years). Informants were predominantly friends (28.4%), parents (14.2%), children (14.2%), spouses (10.7%) or siblings (10.4%) of the participants.

For the extended sample comprising 182 participants, we received informant-reports from 366 informants (average of 2.5 informant-reports per participant). We received three informant-reports for 111 participants (61.0%), two informant-reports for 44 participants (24.2%), and one informant-report for 27 participants (14.8%). The majority of informants was female (283, 63.2%; one informant did not disclose their gender), informants were on average 45.3 years old (SD = 18.0, range = 16–91 years), and had an average relationship duration with the participant of 22.7 years (SD = 16.0, range = 0.15–73 years). Informants were predominantly friends (28.3%), parents (15.0%), children (13.2%), spouses (10.7%) or siblings (10.0%) of the participants.

Behavioral measures. During the lab session, participants completed three behavioral measures: the Balloon Analogue Risk Task, a Delay Discounting paradigm, as well as a mixed gambles lottery task (Supplementary Figure 1).

Balloon Analogue Risk Task. The BART is a computerized behavioral measure structured to capture risk preference in a simulated setting (Lejuez et al., 2002). Although methodological issues resulting from the task's design have been raised (De Groot, 2020; Steiner & Frey, 2021), the BART remains one of the most widely used behavioral measures of risk taking, thus for comparability, we also included it in this study. In the current study, participants' goal was to accumulate monetary winnings by pumping up a series of virtual balloons as much as possible without causing the balloons to explode. Successful pumps earned participants money whereas explosions resulted in the loss of earnings accumulated for the current balloon. Importantly, participants were not told about the balloon capacity or explosion points but rather had to learn and build a mental representation of these contingencies from experience over time. Participants were only told that the balloon could explode at any point between the first pump and the balloon filling up the computer screen. For each successful pump, that is, a pump that did not lead to the balloon exploding, participants earned 0.05 Swiss francs, with earnings accumulating across successful pumps (for example four pumps would earn the participant 0.20 Swiss francs). If participants decided to stop pumping before the balloon exploded, their accumulated earnings (e.g., 0.20 Swiss francs after four pumps) from that particular balloon were "cashed out" and saved in a permanent account. However, if participants continued to pump the balloon leading to an explosion, all accumulated earnings for that particular balloon were lost and no additional earnings were saved to their permanent account.

During the laboratory session, participants completed a total of 40 balloons: 20 balloons with a maximum capacity of 64 pumps and 20 balloons with a maximum capacity of 128 pumps (Supplementary Figure 1, panel a for a schematic of two trials). Participants were informed that there were two balloon types indicated by red and blue colors (colors were randomized between the two capacity conditions across participants) but were not informed about the exact capacities. For any given balloon, the actual explosion point was drawn from a uniform distribution between one and the maximum capacity of that balloon type. Feedback was provided on the screen between trials (that is, at the end of a balloon), informing participants about their accumulated earnings for the current trial (i.e., balloon), and their total earnings after the current trial. We did not manipulate or control the (sequence of) explosion points

between participants. Participants received their accumulated earnings in cash at the end of the lab session.

Delay Discounting. To capture individual differences in impulsive choice (Dalley & Robbins, 2017; MacKillop et al., 2016), we administered a delay discounting task following standard implementation procedures (Eppinger et al., 2012; McClure et al., 2004; Seaman et al., 2018, 2022; van den Bos et al., 2014). Participants encountered repeated binary forced-choice problems between a smaller–sooner or larger–later monetary amount (Supplementary Figure 1, panel b). Participants completed two training trials and 80 test trials, always deciding between a smaller–sooner and larger–later monetary amount. All participants completed the same 80 trials but in randomized order. The 80 trials were based on three levels of a sooner option (today, in two weeks, or in four weeks), and three levels of a later option (in two weeks, in four weeks, or in six weeks). From these levels, we constructed five unique temporal pairings: Today versus in two weeks, today versus in four weeks, in two weeks versus four weeks, in two weeks versus six weeks, and in four weeks versus six weeks. For each of the five unique temporal pairings, we constructed 16 trials based on eight possible percentage differences (1, 3, 5, 10, 15, 25, 35, 50) between the magnitude of the smaller versus larger option. We generated the 80 different magnitudes by drawing 80 random numbers from a normal distribution with a minimum of five and a maximum of 40 (these were the smaller amounts), and, within a given delay-pairing, added the required percentage differences to the smaller amount to yield the respective larger amount.

Performance was incentive-compatible; participants were instructed that one of the trials encountered during task completion would be drawn at random, and their choice realized. If the drawn choice included a delayed option, participants received the amount after that exact delay, that is, we matched the waiting time to the chosen waiting time. For payments at a later time point, participants received the money in cash via registered post.

Mixed Gambles. In addition and contrast to experience-based risk preference captured by the implemented BART version (Schonberg et al., 2011), participants also completed a series of forced choices between two described lotteries (Supplementary Figure 1, panel c), each varying with regards to the number, magnitude and probability of possible monetary outcomes (Tom et al., 2007; Zank, 2010). Participants completed a total of 210 trials between two gambles; the individual gambles presented during each trial were constructed by one author (D.K.) to optimize the computation of model-based indices of loss aversion (i.e., λ). The 210 trials, completed in two runs of 105 trials with a short break in between, were based on 30 trials

for each of seven unique gamble types, each type offering a particular combination of the number, magnitude, and probability of the shown outcomes. For one gamble type, participants decided between two gambles, each with a 50% chance of a certain gain and a 50% chance of a loss of the same magnitude as the gain (e.g., Option A: 58 Swiss francs with 50% and -58 Swiss francs with 50%; Option B: 72 Swiss francs with 50% and -72 Swiss francs with 50%). For another type, each of the two gambles consisted of three outcomes, but all three outcomes were equally likely (i.e., 33.3%) or happened with varying probabilities (e.g., 80%, 10%, and 10%). All outcome probabilities were the same for the two gambles, and the only difference was the magnitude of the two or three possible outcomes. Furthermore, when gambles with two outcomes were presented, each gamble offered symmetric gains and losses (i.e., the same gain or loss magnitude). For gambles with three possible outcomes, two outcomes were symmetric gains and losses, while the third option was the same for the two gambles.

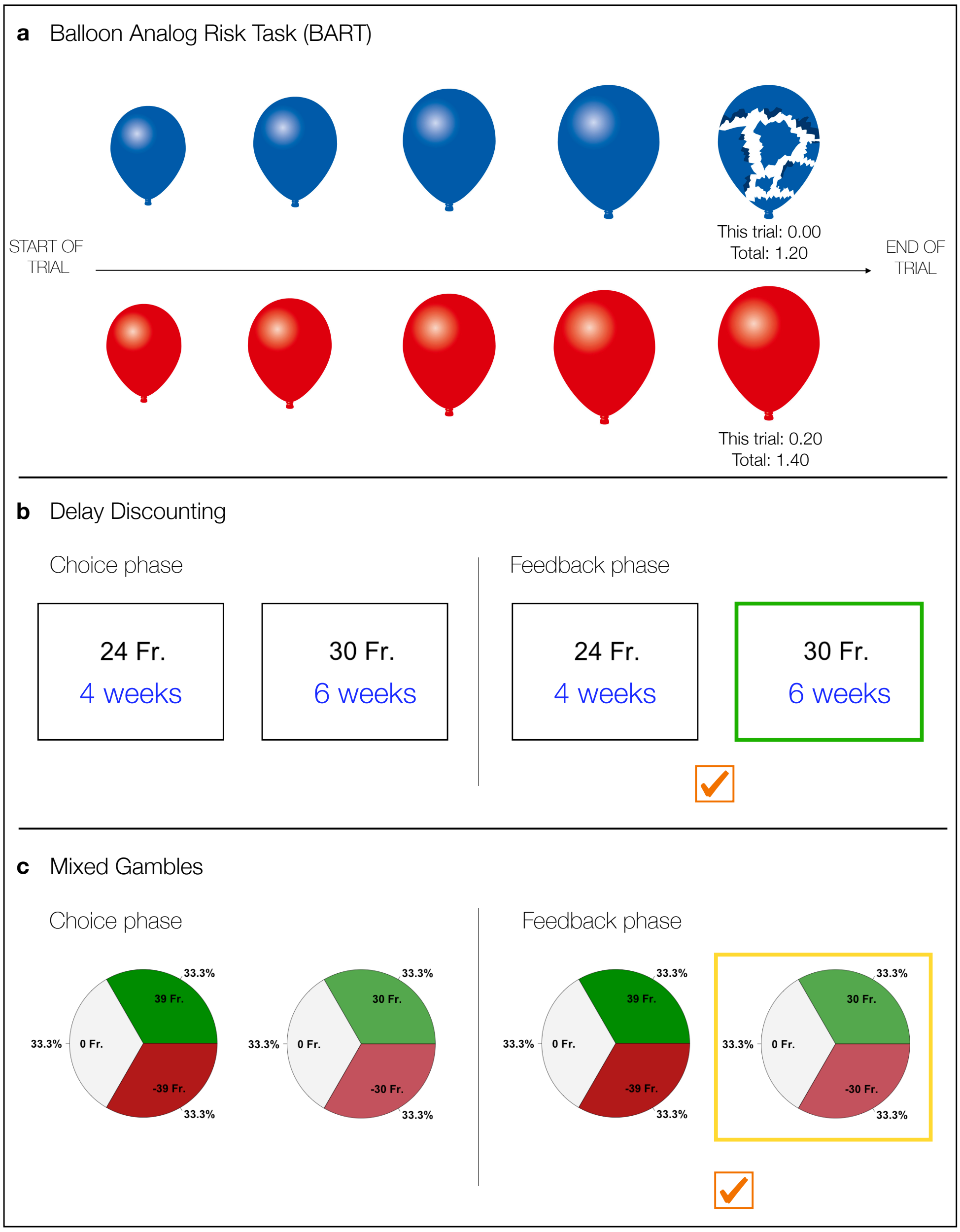

Supplementary Figure 1. Schematic representation of behavioral measures administered during the lab session. (a) Two BART trials, one for a blue balloon that ends up bursting because the participants pumped over the explosion point (critical action "pump" is marked by the black frame), and one for a red balloon for which the participant stops inflation prior to explosion and cashes out the accumulated earnings (critical action "cash out" is marked by the black frame). (b) During the delay discounting task, participants made repeated choices between "smaller–sooner" and "larger–later" rewards. (c) During the Mixed Gambles task, participants made repeated choices between two gambles with different combinations of the number, magnitude, and probabilities associated with possible outcomes. Fr. = Swiss francs.

Supplementary Table 3. Overview of analysis-relevant measures, organized by construct and modality. We list the main indicators for a given measure and provide key references.

Construct (Variable)	Modality	Measure	Key indicator(s)	Reference
**Age (Predictor)**	SR	Date of birth	Age (in years) at lab session (S1)	-
**Risk preference (Outcome)**	SR	SOEP general risk taking	Single-item response score	(TNS Infratest Sozialforschung, 2014)
	SR	SOEP domain-specific risk taking	Single-item response scores	(TNS Infratest Sozialforschung, 2014)
	SR	DOSPERT	Mean subscale scores (propensity ratings)	(Weber et al., 2002)
	SR	Frequency of risky behaviors	Mean subscale scores (4 weeks, 12 months)	(Frey et al., 2017)
	BEH	BART	Pumping behavior	(Lejuez et al., 2002)
	BEH	Mixed gambles	Proportion risky choices	(Tom et al., 2007)
	IR	SOEP general risk taking	Single-item response score	(TNS Infratest Sozialforschung, 2014)
	IR	SOEP domain-specific risk taking	Single-item response scores	(TNS Infratest Sozialforschung, 2014)
	BIO	BART brain activity	VOI activation extracted from contrast analysis	(Schonberg et al., 2012)
**Impulsivity (Outcome)**	SR	Barratt Impulsiveness Scale	Mean subscale scores	(Preuss et al., 2008)
	SR	UPPS	Mean subscale scores	(Schmidt et al., 2008)
	SR	Sensation Seeking Scale	Mean subscale scores	(Beauducel et al., 2003)
	SR	BIS/BAS	Mean subscale scores	(Strobel et al., 2001)
	BEH	Delay discounting	Choice proportion	(Eppinger et al., 2012)
	IR	Barratt Impulsiveness Scale	Mean subscale scores	(Preuss et al., 2008)

Supplementary Table 3 continued.

	IR	UPPS	Mean subscale scores	(Schmidt et al., 2008)
	IR	Sensation Seeking Scale	Mean subscale scores	(Beauducel et al., 2003)
	IR	BIS/BAS	Mean subscale scores	(Strobel et al., 2001)
	BIO	Delay discounting brain activity	VOI activation extracted from contrast analysis	(Eppinger et al., 2012)
**Low self-control (Outcome)**	SR	Brief Self-Control Scale	Mean score	(Bertrams & Dickhäuser, 2009)
	SR	Low Self-Control Scale	Mean subscale scores	(Seipel, 2014)
	SR	GRIT	Mean score	(Fleckenstein et al., 2014)
	IR	Brief Self-Control Scale	Mean score	(Bertrams & Dickhäuser, 2009)
	IR	Low Self-Control Scale	Mean subscale scores	(Seipel, 2014)
	IR	GRIT	Mean score	(Fleckenstein et al., 2014)
**Gender (Covariate)**	SR	Binary-choice item	Male/Female	-
**Education (Covariate)**	SR	Multiple-choice item	Highest level of education	(Zimmermann et al., 2003)
**Numeracy (Covariate)**	BEH	Abbreviated numeracy scale	Number of correctly solved problems	(Weller et al., 2013)
**Working memory (Covariate)**	BEH	Automated operation span	Number of letters recalled in correct position	(Unsworth et al., 2005)
**Hormone (Covariate)**	BIO	Saliva samples	Mean testosterone	(Kurath & Mata, 2018)

Note: SR = self-report; BEH = behavior; IR = informant-report; BIO = biological measure; VOI = volume of interest.

Hormone data. The main goal of the testosterone component of this study is to help estimate the association between trait-like testosterone levels and age-related differences in risk preference. For this purpose, participants were asked to provide up to six saliva samples over two consecutive days (three samples per day, one sample upon awakening, one sample 30-45 minutes later, and one sample in the evening) to obtain a more reliable measure of trait-like testosterone levels which is seldom done in the literature (Dariotis et al., 2016). To promote sample quality, participants received an in-person demonstration as well as detailed written instructions at the end of the laboratory session on how and when to collect their saliva samples, and to store the samples upon collection. Furthermore, participants were required to document the collection of their saliva samples on a paper form, including the recording of the day, time, and time since the last intake of food or drink for each saliva sample. The samples were collected using salivettes (SaliCap set, IBL Hamburg, Germany) as these were judged to facilitate self-administration. All samples were prelabeled to indicate collection day (day 1 or day two) and sample (sample one, two, or three), and were marked with the participant number. Samples were kept in the study participants' refrigerators and frozen immediately after being returned to the researchers.

Neuroimaging measures. Neuroimaging data were collected a on a Siemens 3T MAGNETOM Prisma MRI system with a 20-channel head coil. At the start of the MRI session, we acquired a structural T1-weighted scan for every participant via a magnetization-prepared rapid gradient echo sequence (repetition time = 2,500 ms, echo time = 4.25 ms, inversion time = 1,100 ms, flip angle = 7°, field of view = 256 mm × 256 mm, 192 slices, voxel dimensions = 1.0 mm isotropic). For the task-related functional runs we acquired T2*-weighted blood–oxygen-level-dependent imaging (BOLD) echo-planar images for every person (repetition time = 2,010 ms, echo time = 30 ms, flip angle = 78°, field of view = 192 mm × 192 mm, voxel size = 3 mm × 3 mm × 3 mm, 33 transversal slices per volume with 15% distance factor). Inside the scanner, participants completed two behavioral measures, namely functional magnetic resonance imaging (fMRI) versions of the BART and Delay Discounting.

fMRI BART. The BART has been used extensively in neuroimaging studies to capture the neural correlates of different aspects of risk-taking, including risk, reward, and value integration (Helfinstein et al., 2014; Rao et al., 2008; Schonberg et al., 2012; Tisdall et al., 2020), as well as the respective age effects (Tannou et al., 2021). Here we sought to derive neural markers of risky choice from the BART and to examine age effects on those markers.

Inside the MRI scanner, we ran an adapted version of the BART with three types of balloons: two types (red and blue, counterbalanced across participants) of reward balloon and a gray motor control balloon. Contrary to the BART version implemented in the lab session, the two types of reward balloons in the fMRI version did not differ in their maximum capacity but in the reward functions which determined reward accumulation over time (Supplementary Figure 2). Concretely, both reward balloon types featured explosion points drawn from a uniform distribution with a minimum of one and a maximum of 16 but differed with regards to the payoff function (linear and exponential) underlying reward accumulation for successful pumps. Gray balloons only served as a motor control function for the fMRI contrast analyses and did not contribute to participants' earnings. Participants completed two runs of the BART inside the scanner, each run lasting for approximately 10 minutes. Performance on the task was self-paced and incentive compatible, with earnings being paid out in cash at the end of the fMRI session.

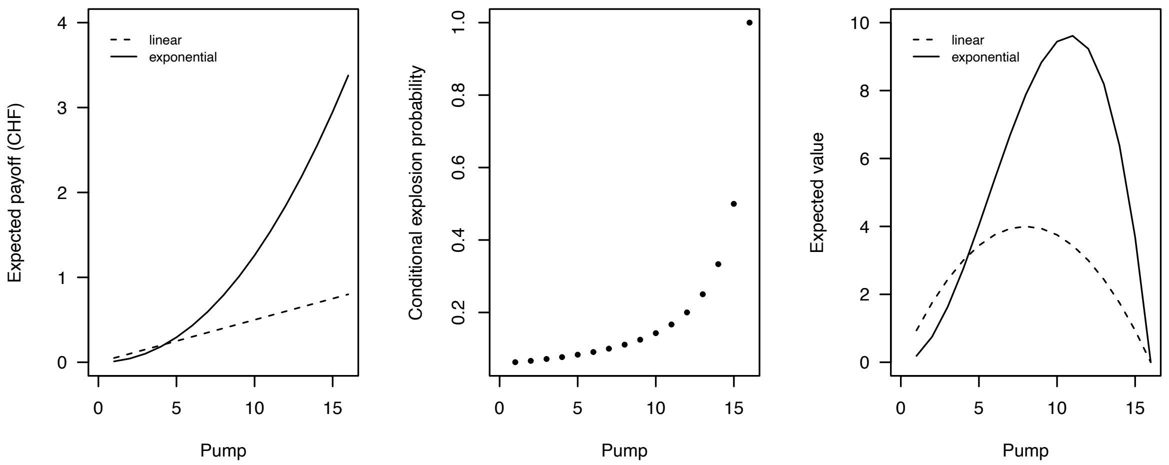

Supplementary Figure 2. Risk and reward in the fMRI version of the BART. CHF = Swiss francs.

fMRI Delay Discounting. Delay discounting paradigms inside the MRI scanner have typically been used to isolate and characterize neural activation differences associated with impulsive choice (Dalley & Robbins, 2017; McClure et al., 2004) as well as age-related differences therein (Eppinger et al., 2012; Samanez-Larkin & Knutson, 2015; Seaman et al., 2018). In this study we followed this approach, aiming to examine age effects on neural functional markers of impulsive choice. As in the laboratory session, we elicited participants' preferences for smaller–sooner versus larger–later rewards. The trial number and structure were identical to the lab session; participants completed 80 trials based on five unique delay-

pairings, with the differences between smaller and larger amounts mapping onto eight different percentage differences. To avoid repetition, the exact magnitudes associated with smaller and larger rewards were however different from the lab session. The fMRI version followed a controlled temporal structure (intertrial intervals between 1 and 11 seconds, mean = 4.32 seconds). Performance was incentive-compatible, and payment was organized the same way as in the lab session (i.e., paid out at the participant-selected delay).

Covariates. Gender is a robust correlate of (self-reported) risk preference (Frey et al., 2021), hence we collected categorical information about individuals' self-reported sex. Furthermore, individual differences in education are associated with risk preference (Frey et al., 2021), although heterogeneity in association patterns highlights the role of both risk preference domain and operationalization. We thus collected data on participants' self-reported highest level of education. It has furthermore been suggested that the divergence of age effects on (especially behavioral) measures of risk preference may be driven by different (behavioral) measures requiring idiosyncratic cognitive processes, such as monitoring and updating processes (e.g., BART) or the integration of numerical information (e.g., Mixed Gambles and Delay Discounting) (Mata et al., 2011; Olschewski et al., 2018). For this reason, we assessed participants' numeracy (Weller et al., 2013), which required the mental computation of frequencies and probabilities for a series of eight specific numerical scenarios or events. We also included the Automated Operation Span task (Unsworth et al., 2005) to capture individual differences in working memory during the recall phase of each trial. Both measures were adopted to capture aspects of individual differences in fluid cognitive capacity.

Data preprocessing

Study completions, missing data, and samples used for analysis. Of the 200 participants recruited for the study, 200 (100%) completed the laboratory session. One participant had to be excluded from all subsequent analyses due to incomplete behavioral measures. 192 study participants (96%) completed the home session, and 189 (94.5%) completed the neuroimaging session. We received 471 informant-reports for a subset of 193 (96.5%) study participants, with one informant-report received for 31 (16.1%), two informant-reports for 46 (23.8%), and three informant-reports for 116 (60.1%) study participants (mean = 2.4 informant-reports). Quality assurance measures revealed incomplete informant-report data for two participants, resulting in a complete set of informant-reports for 191 study participants. Regarding the collection of hormone data, 190 (96.5%) study participants deposited 1,134 saliva samples; we received four

samples from one (0.5%), five samples from four (2.1%), and all six samples from 185 (97.4%) participants (mean = 5.7 saliva samples). To perform analyses on complete data sets while retaining as many participants as possible, we created two inclusive samples. The main sample comprised 148 participants for which we obtained data for all sessions and measures, including biomarker data from the neuroimaging session and saliva samples (see section on Preprocessing of fMRI data for further details about exclusions). The extended sample comprised 182 participants for which we were able to collect all but the biomarker data. The two samples were comparable with regard to their respective demographic characteristics (Supplementary Figure 3).

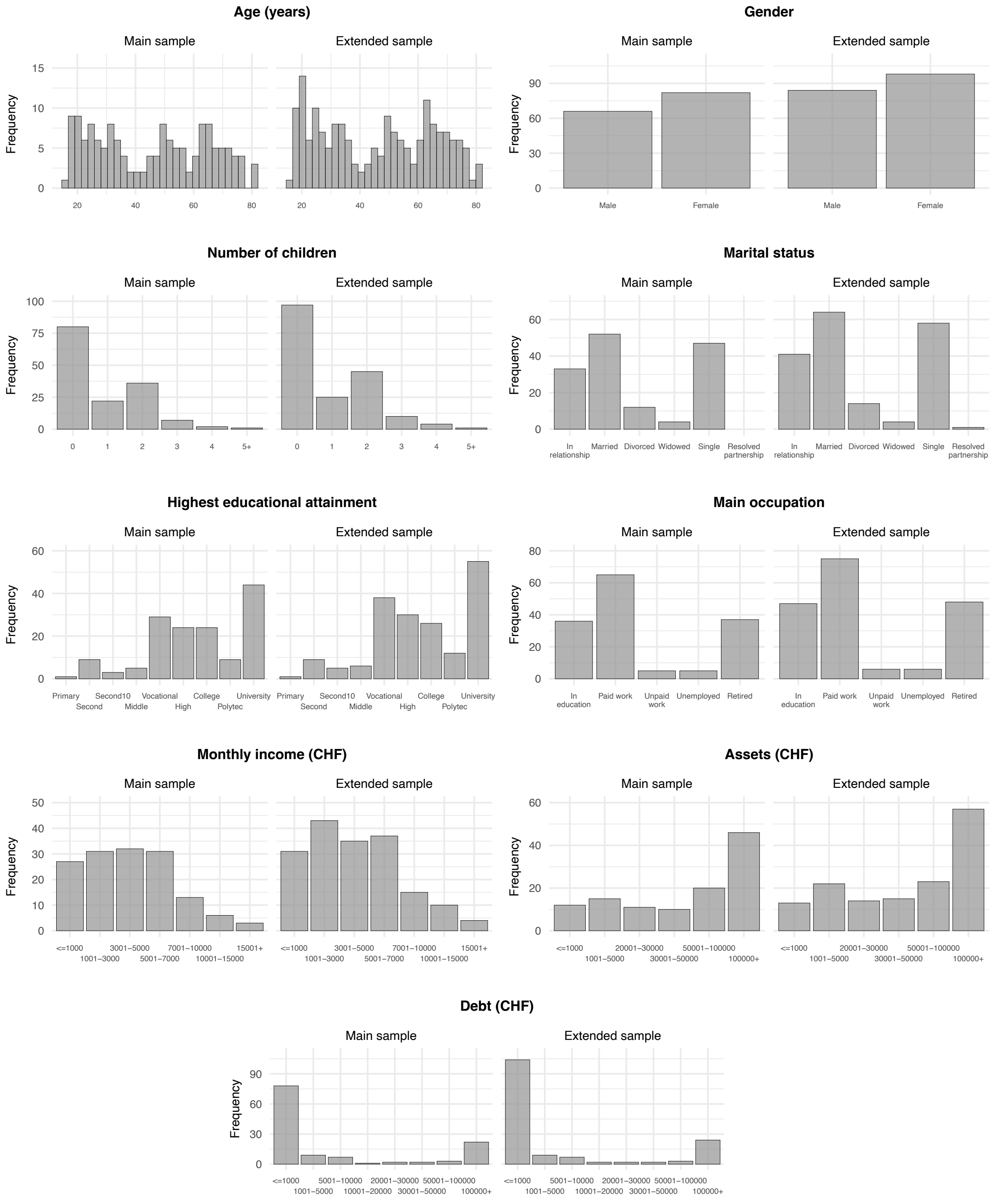

Supplementary Figure 3. Demographic characteristics for main sample (N = 148) and extended sample (N = 182). The two samples were comparable across all demographic and socio-demographic variables.

Dimensionality reduction of self-report data. As detailed in our analysis plan and preregistration, we planned to perform a confirmatory factor analysis informed by previous work (Eisenberg et al., 2019; Frey et al., 2017) to extract latent variables from the battery of

self-reports capturing individual differences in risk preference, impulsivity, and low self-control. Unfortunately, this confirmatory factor analysis approach via implementation of a bifactor model indicated that the model fit was unsatisfactory, both for the main sample (N = 148) [comparative fit index (CFI) = 0.72; Tucker–Lewis index (TLI) = 0.67; root mean square error of approximation (RMSEA) = 0.10; standardized root mean square residual (SRMR) = 0.11; df = 371] and the extended sample (N = 182) [comparative fit index (CFI) = 0.73; Tucker–Lewis index (TLI) = 0.68; root mean square error of approximation (RMSEA) = 0.09; standardized root mean square residual (SRMR) = 0.11; df = 371].

Given that our sample size placed considerable restrictions on alternative analytical methods to gauge and extract model-driven indices of risk preference, we deviated from our analysis plan and opted for dimensionality reduction of self-report measures by comparing composite scores extracted using either unit-weighting or principal component analysis. Both approaches involved the same four initial preprocessing steps. First, all single self-report items were coded so that higher scores indicated higher risk preference, higher impulsivity, or lower self-control. Second, where items could be aggregated to the level of subscale scores (e.g., DOSPERT domain-specific risk preference scales), we averaged across single items to derive subscale scores. This procedure resulted in 32 subscale means for every participant. Third, we handled missing data through exclusion and imputation. Due to the distribution of measures across sessions, participants had either no (all self-reports completed, n = 191), five (lab and home self-reports completed, n = 1), or a large number (>20) of missing subscale scores (e.g., only self-reports from the lab session completed, n = 8). We excluded the eight participants with more than 20 missing subscale scores. For the single participant with five missing subscale scores, we used the R package *mice* to impute missing variables with the number of multiple imputations set to one, using a predictive mean matching algorithm with 50 iterations. The procedure was seeded to allow for replicable imputation. Fourth, we assigned the 32 self-report subscales to one of three constructs (that is, whether a self-report was selected to capture risk preference, impulsivity, or low self-control) and computed bivariate correlations between scores within a given construct to check for consistent coding and convergence of subscale measures within a construct.

In both the main and the extended sample, self-report scores were mainly positively correlated within the respective constructs of risk preference, impulsivity, and low self-control (Supplementary Figure 4). We thus proceeded with the computation of construct-specific composite scores. For the unit-weighting approach, we computed construct-specific scores by

summing participants' standardized (within subscale) responses for a given construct. We also computed one unit-weighted score across all self-report subscales to mirror the idea of general versus construct-specific individual differences. For comparison, we pursued a similar yet psychometrically more principled approach and performed a principal component analysis using the built-in R function *prcomp*. Specifically, we used the same construct-specific groupings of self-report variables and extracted, respectively, the first principal component for risk preference, impulsivity, and low self-control. We also extracted a construct-independent index by extracting the first principal component across all self-report measures of risk preference, impulsivity, and low self-control. Given the very high correlations between individuals' composite scores derived via the two approaches (mean $r_{\text{Pearson}} = 0.98$ for both main and extended samples), we continued our analyses using the model-free unit-weighting scores as these are easy to interpret.

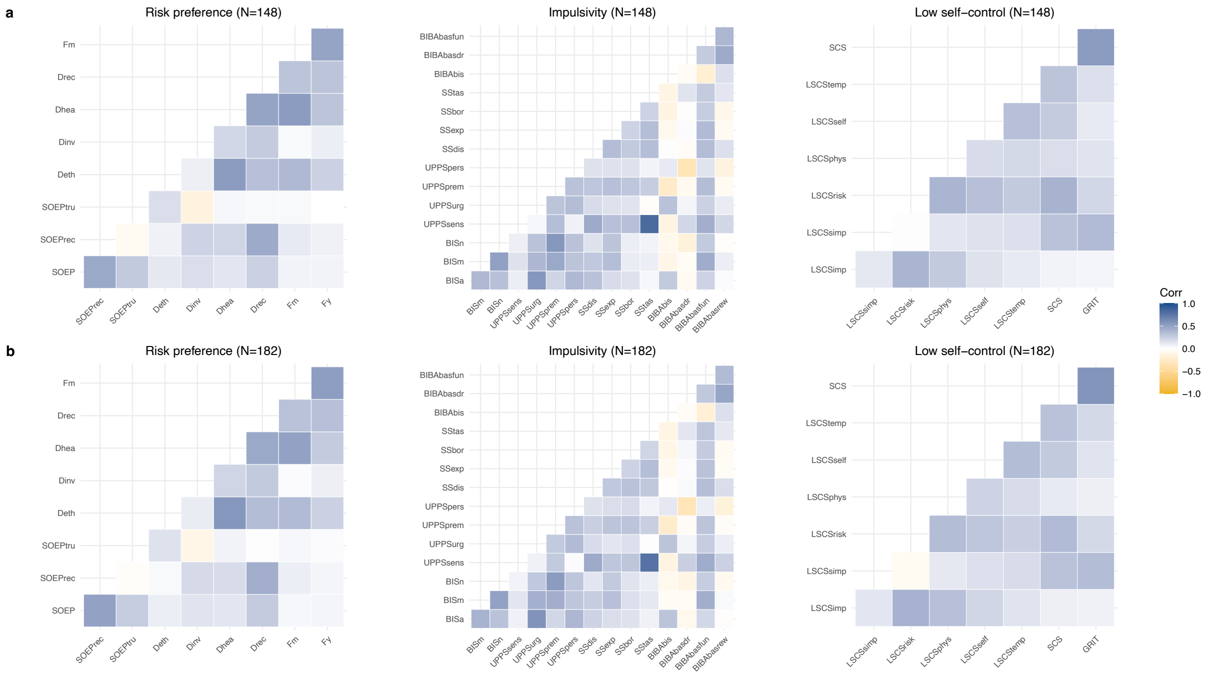

Supplementary Figure 4. Associations between (sub)scale scores from self-report measures, organized by construct. (a) Pearson correlations for the main sample. (b) Pearson correlations for the extended sample.

Dimensionality reduction of informant-report data. We pursued the same approach for the reduction of informant-report data as described for the self-report measures. For the informant

data, we also observed high correlations between informants' construct-specific unit-weighted scores and the first principal component extracted for each construct (mean $r_{Pearson}$ = 0.99 for the main and extended sample, Supplementary Figure 5). As for the self-report measures, we continued our analyses with the model-free unit-weighted indices, that is, a general informant-report index, and three indices specific to measures of informant-reported risk preference, impulsivity, and low self-control.

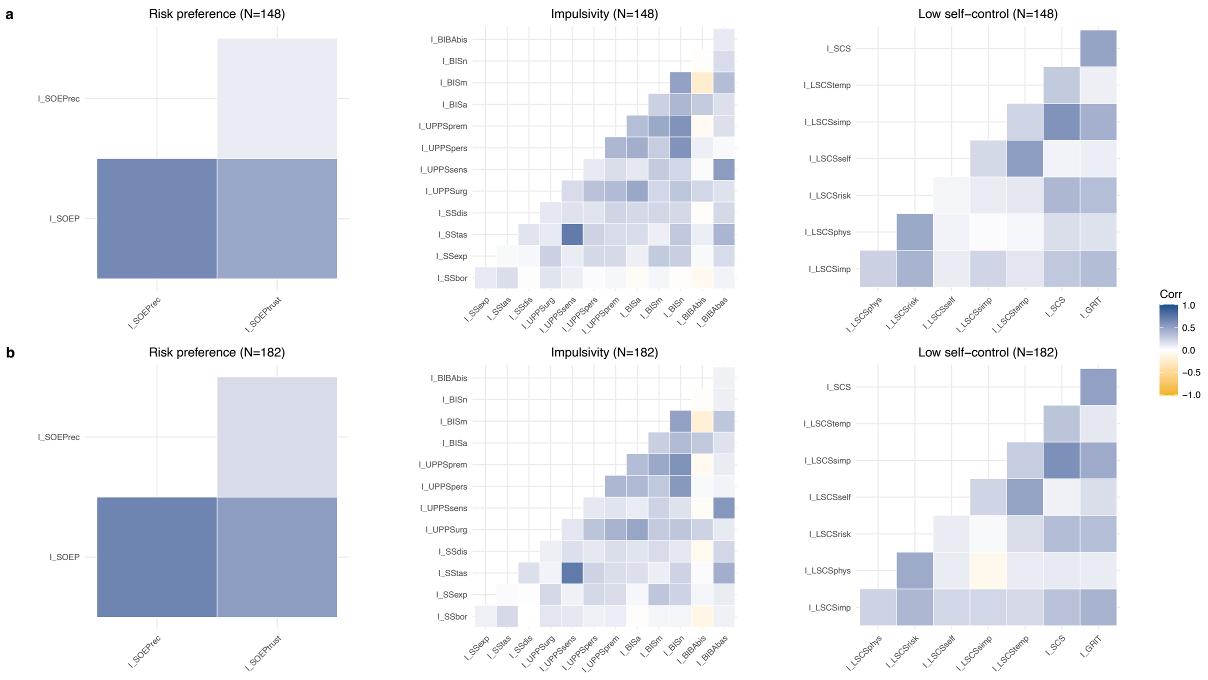

Supplementary Figure 5. Associations between (sub)scale scores from informant-report measures, organized by construct. (a) Pearson correlations for the main sample. (b) Pearson correlations for the extended sample.

Performance-based indices from behavioral measures. Data from the behavioral measures collected during the lab session were preprocessed and performance-based indices were extracted by individual analysts. As part of our planned analyses, we explored BART performance indicators and fitted computational models to the Delay Discounting and Mixed Gambles data.

BART. Recent research has suggested that common BART scores may not be ideal to capture interindividual differences in people’s (trait) risk preferences (De Groot, 2020; Steiner & Frey, 2021). To further explore any age-specific associations along these lines, we first

computed three indices as descriptive measures of the behavior in the BART (separately for the two types of balloons that were implemented): 1) The average number of pumps across trials of the same balloon type (Lejuez et al., 2002), the adjusted number of pumps across trials of the same balloon type (Lejuez et al., 2002), and 3) the total number of explosions across trials of the same balloon type (Schmitz et al., 2016). As planned, we inspected the correlations between these indicators and, as expected, found them to be highly correlated (Supplementary Figure 6, panel a). Following plans laid out in the analysis plan, we subsequently only used the adjusted number of pumps as an indicator of risk preference in the BART. Moreover, the adjusted number of pumps for balloon types with different capacities was $r_{Pearson} = 0.58$, hence we collapsed the BART indicator across balloon types and proceeded with our main analyses using only one BART indicator.

Although sophisticated computational models have been developed for the BART (Park et al., 2019; Pleskac, 2008; Pleskac & Wershbale, 2014; Wallsten et al., 2005), there exists an ongoing debate concerning the extent to which model parameters provide additional insights over the directly observable outcome measures, and/or the extent to which different parameters are sufficiently independent and recoverable (van Ravenzwaaij et al., 2011). For this reason, we exclusively report the directly observable outcome measures as detailed in the previous section. However, to visually assess the extent to which learning effects may play a role, we plotted the adjusted number of pumps as a function of trial index (Supplementary Figure 6, panel b). As expected, indices were highly correlated, justifying the use of one indicator for the main analyses. Moreover, no learning effects were discernible, as the mean number of adjusted pumps (averaged across participants) did not change over trials, and did also not change as a function of trial type.

Delay discounting. We calculated two measures of delay discounting based on the 80 decision problems presented to each individual. First, we calculated the proportion of sooner smaller choices. Second, we used the basic hyperbolic discounting model (Mazur, 1987) to determine the discounting factor *k*. To achieve this, we used a maximum likelihood approach recruiting a power choice rule (Wulff & van den Bos, 2018) and the Nelder–Mead optimization algorithm. We observed that for 14 out of 200 individuals, the hyperbolic discounting model did not account for choices better than an intercept-only base model. Furthermore, we observed that for seven individuals discounting factors in excess of 1 were estimated, which also signals model misspecification. For these reasons and for the fact that the proportion of sooner smaller choices and the discounting factor *k* were highly correlated ($r_{Spearman} = 0.98$), we focused our main analyses exclusively on the proportion of sooner smaller choices.

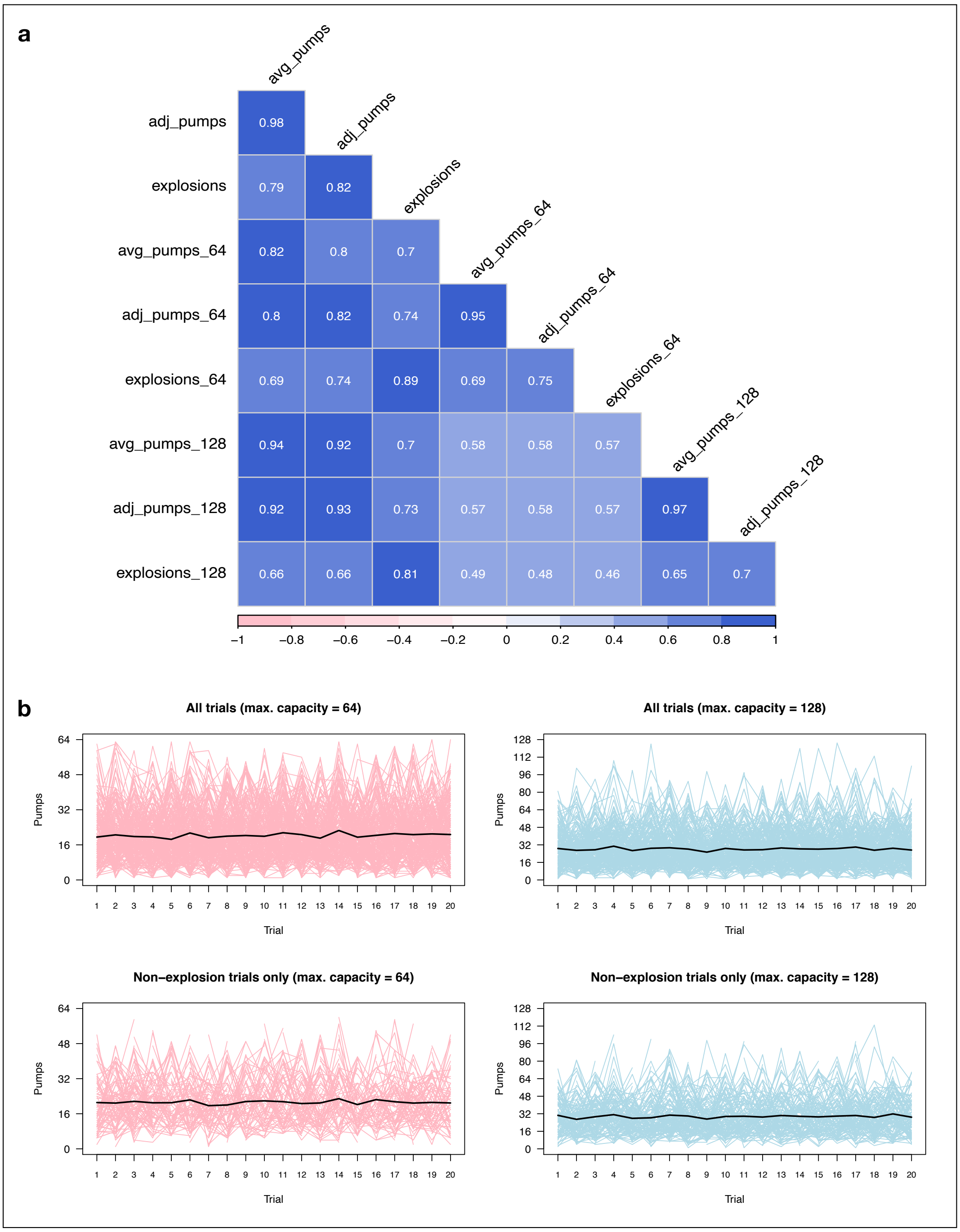

Supplementary Figure 6. BART performance indices. (a) Correlation coefficients between different performance indicators were computed for all participants for which BART performance data was collected during the lab session (n = 199). adj_pumps = mean number of pumps across nonexplosion trials; avg_pumps = average number of pumps across all trials. (b) Pumping did not change over time, suggesting no substantial learning effect across BART trials.

Mixed gambles. The main statistic of interest was the proportion of risky choices, P(risky), across 210 trials, where the "risky option" was the lottery with greater outcome variance. Given the lotteries used (symmetrical mixed lotteries with Expected Value = 0), we expected P(risky) to track the Prospect Theory parameter lambda as well as any kind of gain-loss asymmetry at the level of probability weighting [see (Zank, 2010) for a theoretical rationale]. As an auxiliary measure, we estimated the loss-aversion parameter λ, by fitting a Prospect Theory model to the choice data. The specific Prospect Theory model assumed linear utility, nonparametric probability weighting, and a logistic choice function. Asymmetries between gain and losses would exclusively be captured by λ. The motivation behind the specific model was that it provides a competent and parsimonious characterization of the data. This was especially important in the present case given that the choice data concerned a very specific kind of lottery (symmetrical mixed lotteries with Expected Value = 0), limiting the ability to estimate parameters. The reason for the nonparametric estimation of probability weighting was that all nonzero outcomes in the lotteries were associated with one of three possible probability values (1/10, 1/3, and ½). The weighting of such value was done by setting up three weights $0 \leq w1 \leq w2 \leq w3 = 1$ without a loss of generality. This weighting scheme established the same number of free parameters as the most common parametric weighting functions adopted (e.g., GoldsteinEinhorn). The performance-based index P(risky) was highly correlated with the loss-aversion parameter λ ($r_{Spearman} = -0.86$), thus we performed all our main analyses using the proportion of risky choices.

As part of the peer review process, we performed additional (i.e., unplanned) analyses to examine possible trial effects in the mixed gambles task. Mixed-effects modeling revealed a small but significant effect of trial number on choice (log odds = -0.05, SE = 0.01, $p < 0.001$), such that individuals became less risk seeking over time. There was no interaction between trial number and age (log odds = 0.00005, SE = 0.01, $p = 0.99$). However, visualization of the trial effect (Supplementary Figure 7) revealed that this effect was driven by the first ~25 trials, after which choice patterns remained relatively stable for the rest of the 210 trials. The results were comparable for the main sample and the extended sample. Considering the minimal scope and impact of this effect, we retained the original performance indices used for this task in our analyses.

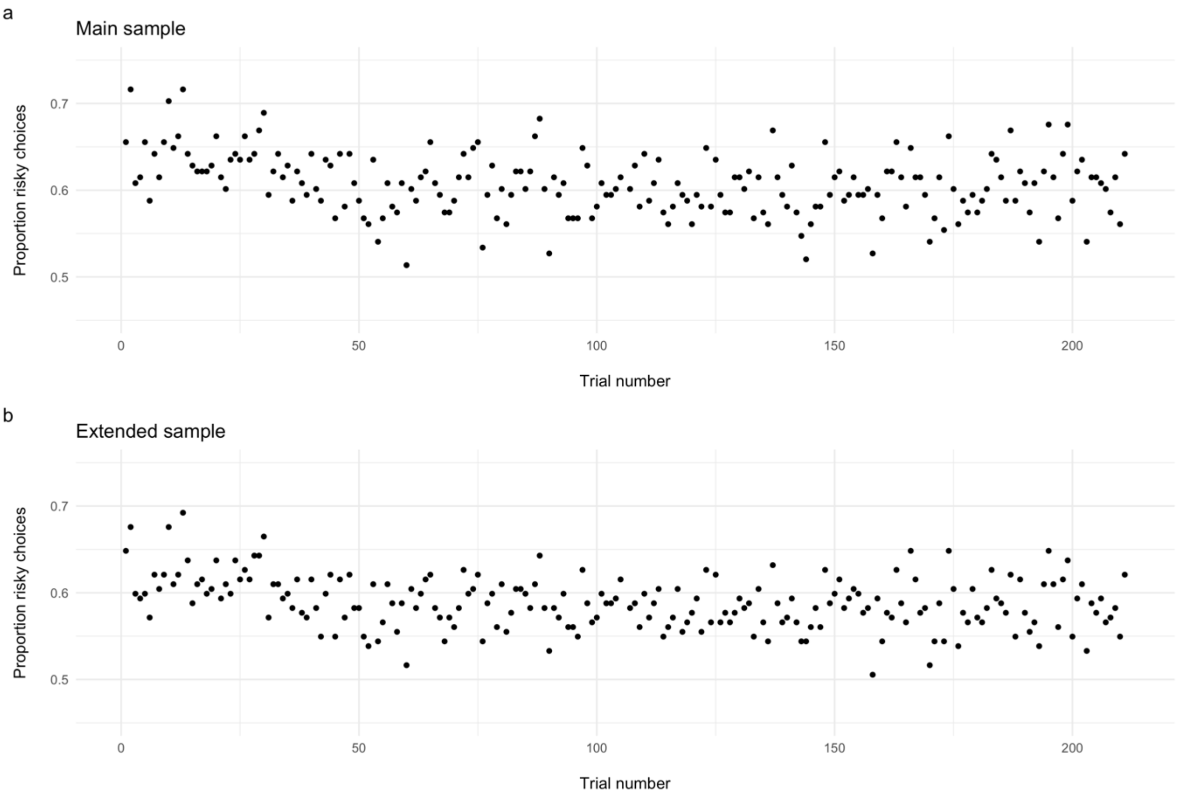

Supplementary Figure 7. Proportion of risky choices over time in the mixed gambles task for (a) the main sample, and (b) the extended sample.

Preprocessing of hormone data. Upon the completion of data collection, participants' saliva samples were batch analyzed at an independent external lab (http://www.dresden-labservice.de) given their expertise and suitable equipment for the assaying of saliva samples for testosterone. The frozen samples were shipped on dry ice to ensure sample integrity during transport. After quality control by the external lab, we received 1134 testosterone scores (unit = picograms/milliliter) for 190 participants, that is, between four and six measurements per participant. We obtained a record from the external laboratory of all samples received, the quality of each sample, as well as instances of samples that were double-checked to ensure individuals' testosterone data were valid. Post assaying, all samples were destroyed by the lab.

Concerning the preprocessing of the individual-level hormone measures obtained from the lab, past work on basal testosterone levels is heterogeneous in the methods used to preprocess hormonal data (Kurath & Mata, 2018; Pollet & van der Meij, 2017), including the use of different procedures that attempt to deal with measurement error via the removal of outliers or winsorizing (Harden et al., 2018; Pollet & van der Meij, 2017). We thus opted to not perform any transformations (e.g., log) because any rationale for transformation is unclear

and no transformation is the modal method (ca. 60%) in the testosterone and risk preference literature (Kurath & Mata, 2018). Concerning outliers, we followed recommendations to conduct sensitivity analyses to assess the effects of outlier selection (Pollet & van der Meij, 2017). Specifically, we checked whether winsorizing outliers that deviate at least three (sex-specific) standard deviations from the (sex-specific) mean provided considerably different estimates of average testosterone level relative to no processing of outliers. The Pearson correlation between the estimates with and without preprocessing of outliers (winsorizing) was very high, $r_{Pearson}$ = 0.99, $CI_{95\%}$ [0.98, 0.99], hence we continued our analyses without preprocessing the testosterone estimates, and report only the simpler index (i.e., average per participant without preprocessing of outliers). That is, we produced one index that represents overall individual differences in testosterone levels by averaging testosterone levels across all available measurements per participant.

Preprocessing of covariate measures. To capture individual differences in numeracy based on participants' responses to eight numerical problems (Weller et al., 2013), we calculated an index based on the total number of correctly solved problems, yielding a score from zero (no problems solved correctly) to eight (all problems solved correctly). To capture working memory based on the recall stages of the automated operation span task (Unsworth et al., 2005), for each participant we calculated an index based on the total number of letters recalled in the correct position across all trials, yielding a score between zero (no letters recalled in the correct order) and 75 (all letters recalled in the correct order).

Neuroimaging data analysis

We collected neuroimaging data for 189 participants, 11 were lost to follow-up between the lab and MRI session (Figure 1). For 10 of the 189 participants, we were unable to collect functional runs for both behavioral measures (mainly because we were unable to run delay discounting due to time constraints at the scanner facility) leading to their exclusion from the imaging analyses, and a further two participants were excluded due to faulty scanning equipment leading to image artifacts. Preprocessing of the raw data, as well as individual- and group-level contrast analyses were performed using standard statistical routines implemented in SPM12 (https://www.fil.ion.ucl.ac.uk/spm/software/spm12/). Coordinates are reported in MNI space (mm). To visualize group-level activation differences on a standard group template in MNI standard space, we used the Multi-image Analysis GUI Mango (https://ric.uthscsa.edu/mango/mango.html).

Preprocessing of fMRI data. Participants' functional runs from the BART and Delay Discounting were preprocessed as follows. First, individuals' functional volumes were spatially realigned via a two-pass procedure to the series' mean image, and realignment parameters for six directions (three rotation, three translation parameters) were estimated and saved. Second, we adopted standard slice time correction methods to account for the interleaved (bottom-to-top) acquisition of the fMRI volumes. Third, individuals' spatially and temporally realigned functional volumes were coregistered to their structural volume via maximization of a normalized mutual information objective function. Fourth, we segmented individuals' anatomical images and then used the information from the segmentation procedure to normalize (warp) the functional volumes from native to standard Montreal Neurological Institute (MNI) space via individuals' anatomical volumes. In the fifth and final step, the realigned, coregistered and normalized functional volumes were smoothed using a 4-mm full-width half-maximum Gaussian kernel (Sacchet & Knutson, 2013) to control for residual anatomical differences between participants.

Given the age-related heterogeneity of the current sample, all functional volumes of all participants were individually inspected to ensure segmentation and normalization procedures had not failed or resulted in misalignment. Normalization was successful for all brains. We also plotted individuals' realignment parameters for all functional volumes, which led to the detection and exclusion of two individuals with excessive head motion (>4mm absolute volume-to-volume translational differences) from subsequent analyses involving fMRI data.

fMRI model specification—input models. After preprocessing, we first performed statistical analyses to estimate the input model, that is, to estimate voxel-wise activation differences for specific time periods in the BART and Delay Discounting.

BART. For the BART, we were mainly interested in extracting key neural markers of decision periods associated with risk taking. For this purpose, for each participant, we first concatenated the two BART runs and the specified one general linear model. The BART general linear model included onset vectors for control balloons and the two reward balloons; preliminary analyses had revealed no activation differences between the two reward balloons (linear versus exponential payoff), thus we collapsed across these two balloon types. For reward and control balloons, we modeled activation differences from the time of trial onset (that is, from the time of balloon display onset) to trial offset (that is, the time after the choice was made and before feedback was received); in essence, the modeled duration was the

stimulus-specific reaction time. We included additional trial-relevant events in the general linear model (e.g., onset vector for explosion events and parametric pump regressors for all balloon types) as well as the six motion parameters estimated during the realignment procedure as regressors of no interest in order to a) achieve a "cleaner" baseline signal and b) control for contrast-irrelevant activation differences.

For the main analysis, we contrasted pumping on reward balloons with pumping on control balloons ("Pumps reward versus Pumps control"), as the latter comprised the motion associated with pumping but not the active decision-relevant mental representation associated with inflating reward balloons (Rao et al., 2008; Schonberg et al., 2012; Tannou et al., 2021). This is a standard contrast often utilized to isolate decision-relevant aspects in varying regions of the brain, including (anticipatory) reward signals and (anticipatory) loss signals (Schonberg et al., 2012; Tisdall et al., 2020). Although the fMRI BART was administered as a self-paced behavioral measure, all participants completed a sufficient number of reward balloon trials (mean = 40.89, median = 40, range = 29–51) to permit the planned contrast analyses.

Although we were not explicitly interested in average activation differences, that is, activation differences at the level of the group (N = 148), we computed a one-sample *t*-test testing whether and where in the brain the average (group-level) signal in response to reward balloon pumps versus control balloon pumps was significantly different from zero. These analyses were only performed to examine whether average contrast-related activation differences matched those reported in the literature, but did not inform or influence any of our output models at the level of individual differences analyses. Group-level activation differences for the BART (Supplementary Figure 8, panel a) were in line with published whole-brain analyses of activation differences for comparable contrast analyses (Schonberg et al., 2012; Tannou et al., 2021; Tisdall et al., 2020).

Delay discounting. For Delay Discounting, we sought to isolate neural markers associated with impulsive choice (Dalley et al., 2011; Eppinger et al., 2012) by contrasting time periods including smaller–sooner choices with choices of larger–later options. At the level of the individual, we specified one general linear model for neural activation differences in delay discounting. As our focus was on contrasting smaller–sooner *choices* with larger–later *choices*, we included one onset vector for trials in which the smaller–sooner option was selected, and a separate onset vector for trials in which the larger–later option was selected, as well as six motion regressors of no interest. As for the BART, we modeled the time period from trial onset (onset of the display showing the two choice options) to trial offset (time after the choice was made and before the onset of the next display). Twelve participants had less

than 10% choice variance, that is, one option (i.e., smaller–sooner or larger–later) was picked eight times or less; these participants were excluded from analyses as it was not possible to robustly compute the planned contrasts for these participants. Given that analyses of delay discounting performance in the laboratory session indicated a high correlation between performance-based (proportion of smaller–sooner choices) and modeling (discounting parameter *k*) indices, we did not include any further model-related variables in the model.

Following the rationale and approach to group-level activation differences in the BART, we computed a one-sample *t*-test to assess whether and where in the brain the average (group-level) signal for smaller–sooner versus larger–later choices was significantly different from zero. Group-level activation differences for Delay Discounting (Supplementary Figure 8, panel b) were comparable to published whole-brain analyses of activation differences for comparable contrast analyses (Eppinger et al., 2012; McClure et al., 2004; Samanez-Larkin et al., 2011).

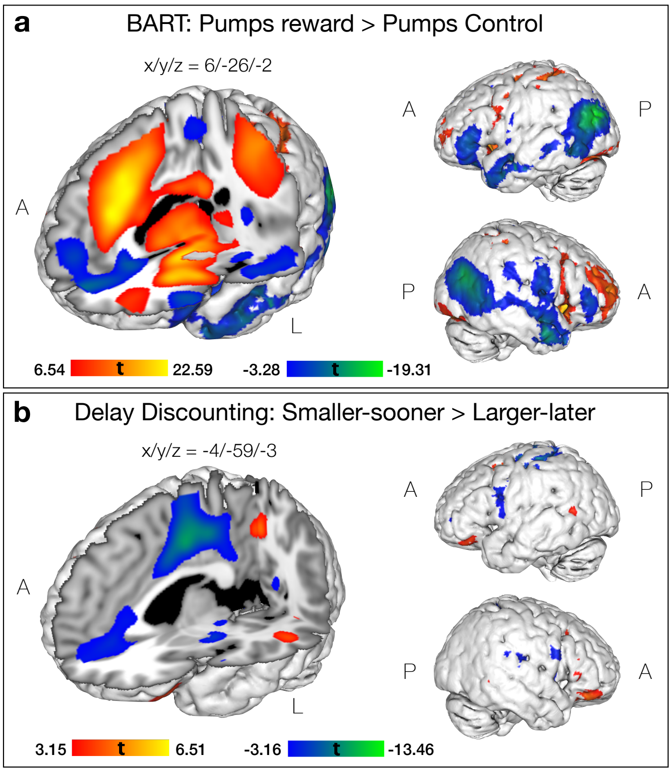

Supplementary Figure 8. Group-level brain activation differences for imaging measures for the main sample (N = 148). (a) BART whole-brain average activation differences for pumps on reward balloons versus control balloons. (b) Delay Discounting whole-brain average activation differences for selecting smaller sooner versus larger later monetary offers. All group-level analyses controlled for head motion, age, and gender; $p < 0.001$, uncorrected. A = anterior, P = posterior, L = left.

fMRI model specification—output models.

Selection of Volumes of Interest. Based on the aggregation and integration of numerous magnetic resonance imaging (MRI) studies, the Affect–Integration–Motivation (AIM) framework (Samanez-Larkin & Knutson, 2015) offers a neurobiological foundation for studying the effect of age on affective and motivational brain circuits supporting risky decision-making. As a starting point, the AIM framework breaks the decision-making process down into various sequential, hierarchically arranged processes identifies their respective neural correlates, and consequently stipulates several pathways for how age-related anatomical and functional change may lead to age-related differences during varying decision-making stages (Frazier et al., 2019; Samanez-Larkin & Knutson, 2015). In essence, AIM proposes as a first stage *affective* anticipation of gains (via projections from the dopaminergic midbrain to the nucleus accumbens) and losses (via projections from the dopaminergic midbrain to the anterior insular cortex), followed by a second stage during which the output of the first stage becomes

integrated into a value signal (via projections to the medial prefrontal cortex), which in turn are converted into a *motivational* signal promoting subsequent (in)action (via projections back to the dorsal striatum and supplementary motor area). The AIM framework provides a set of volumes of interest (VOIs) for the targeted, a priori-defined extraction of neural markers, in particular in the context of age-related differences. Informed by construct-specific target regions (Dalley et al., 2011; Samanez-Larkin & Knutson, 2015), and given statistical constraints with regards to the number of neural variables used as outcome variables in the main analyses, we decided to focus on brain regions with proposed functional relevance both by AIM and construct-specific literature, namely gain anticipation in the nucleus accumbens (BART and delay discounting), loss anticipation in the anterior insula (BART given the experience-based choice format), and medial prefrontal cortex for integrative processes (delay discounting).

*Construction of VOI masks***.** The VOIs were structurally defined based on the probabilistic Harvard–Oxford cortical and subcortical structural atlases (Desikan et al., 2006) rather than functionally defined spheres around peak voxels (Poldrack & Mumford, 2009). In the first step, we saved out masks for the following regions (labels as given in the atlases): "Frontal Medial Cortex", "Left Accumbens", "Right Accumbens", "Insular Cortex", which were thresholded at 20% (thus excluding voxels with a probability of less than 20% of belonging to the anatomical structure). We combined the two nucleus accumbens hemispheres into a bilateral mask. Furthermore, we cut the insular cortex mask at $y = 0$, so that only voxels $y > 0$ were selected for an inclusive anterior insula mask (Samanez-Larkin & Knutson, 2015; Tisdall et al., 2020). In the final step, the three VOI masks were binarized and then used for the extraction of mean beta coefficients from the respective brain regions (Supplementary Figure 9). After plotting the extracted mean beta values for each VOI and each of the two measures, four additional participants were excluded from the main sample analyses because their mean beta values extracted from contrast analyses in any VOI for any fMRI measure exceeded 4SDs.

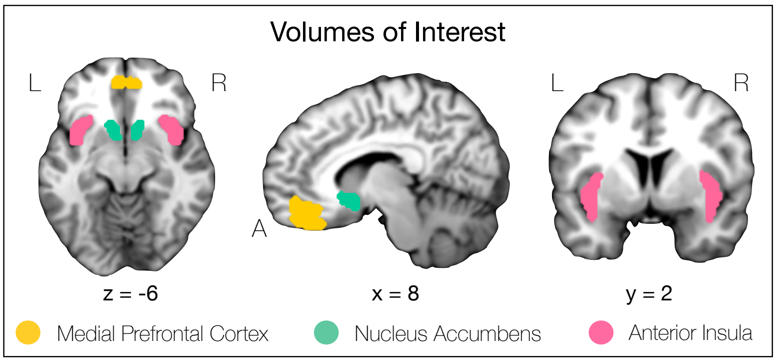

Supplementary Figure 9. Volumes of Interest in neuroimaging analyses. L = left, R = right, A = anterior.

Bivariate associations

Following published guidelines (Millroth et al., 2020), we generated network plots using the R package *qgraph* (Epskamp et al., 2012). We restricted the network plots to associations with an absolute strength of $r_{Pearson}$ > = 0.15, and used a Fruchterman–Reingold algorithm with repulsion parameter of 0.7 to determine the relative spacing of variables in the network based on the strength of their associations.

Multiverse analysis for the main and the extended sample

To examine associations between outcome variables, predictors, and covariates in a comprehensive yet (visually) accessible way for the main sample (N = 148, biomarker data) and the extended sample (N = 182, no biomarker data), we followed a multiverse analysis approach (Steegen et al., 2016) and implemented a Specification Curve Analysis, SCA (Simonsohn et al., 2020). Previously, SCA has been adopted to investigate the robustness of empirically observed associations between variables contingent upon variation in the operationalization of outcome variables as well as the inclusion or exclusion of covariates (Frey et al., 2021; Orben & Przybylski, 2019; Rohrer et al., 2017). Here we used SCA to assess and visualize (variance in) the separate effects of our main predictor, age, on a range of outcomes, that is, different indices of risk preference and related constructs, while controlling for covariates; the "packaging" of all relevant (reasonable, statistically valid and nonredundant) analytical specifications as a set of models thus achieves a principled, exhaustive quantification of this effect.

For the main sample, the total number of specifications was obtained as follows: For each of the 15 outcomes (DVs), we generated all possible additive combinations of the

predictor age (IV) and the five covariates, with age and the outcome always contained in the model but varying numbers of covariates (and their combinations). This additive combinatorial approach resulted in 15*2^5 = 480 unique specifications, each of which we estimated using ordinary least squares regression models using the R package *specr* (Masur & Scharkow, 2020). To quantify the SCA results, we computed the number of positive, negative, and null effects for age (controlling for covariates) on risk-preference indices and related constructs, and report median effect sizes for age.

For the extended sample, the set of specifications was based on one predictor (age), four covariates (sex, education, numeracy, working memory), and 11 outcome variables (four indices from the analysis of self-report measures, four indices from the analysis of informant-reports, and three indices stemming from the behavioral measures). Using the combinatorial additive approach, we generated 11*2^4 = 176 unique specifications for the extended sample. We adopted the same permutation testing routine to ascertain the global significance of the SCA for the extended sample as for the main sample (Rohrer et al., 2017).

Permutation testing

We adopted a permutation-based approach (Rohrer et al., 2017) in order to ascertain the global significance of the observed specification curves. This entailed the creation of 500 versions of the same data set used for the SCA, but with the age variable being randomly sampled with replacement. For each of the 500 shuffled data sets, we performed an SCA. To derive a global significance value for the empirical SCA, we counted the number of SCAs on the sampled data (out of 500) that had a larger number of significant effects than observed for the original SCA performed on the original data. We then divided this number by the number of shuffles, i.e., 500.

SUPPLEMENTARY RESULTS

Bivariate Associations

We computed bivariate correlation coefficients between all variables entering into the multiverse analyses; that is, the predictor, outcome and control variables specified in the Specification Curve Analysis for both the main and the extended sample (Supplementary Figure 10). The correlation coefficients provided the basis for the network plot visualizations.

a

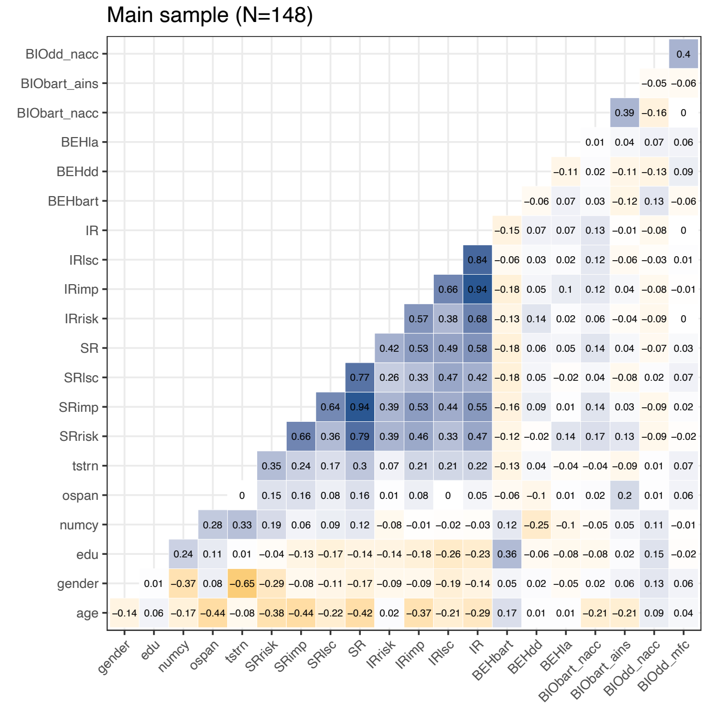

b

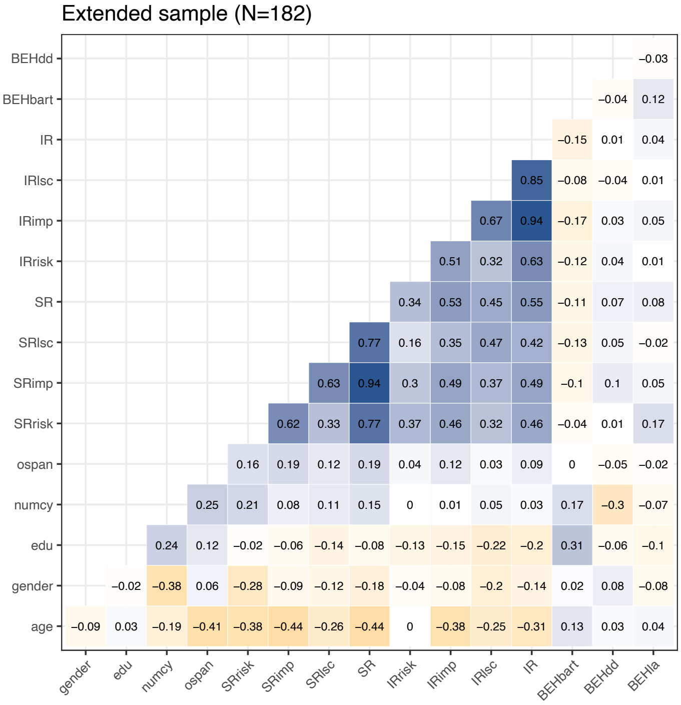

Supplementary Figure 10. Correlation matrices for variables entered into multiverse analyses. (a) Bivariate Pearson correlation coefficients for the main sample (N = 148). (b) Bivariate Pearson correlation coefficients for the extended sample (N = 182). For labels, see Table 1.

Network plot for the extended sample

Bivariate associations between all variables entered into the main analyses for the extended sample mirrored the general patterns observed for the main sample (Supplementary Figure 11). Variable clusters (in terms of higher correlation coefficients) were observed between composite variables based on self-report (SR, yellow), between composite variables based on informant-report (IR, green), as well as between composite self-report and informant-report variables. Moreover, correlations were higher between age and self- or informant-report variables than for age and indices from behavioral measures (blue). As expected, age was negatively associated with cognitive capacity (numeracy and working memory, dark magenta), but not with gender or education.

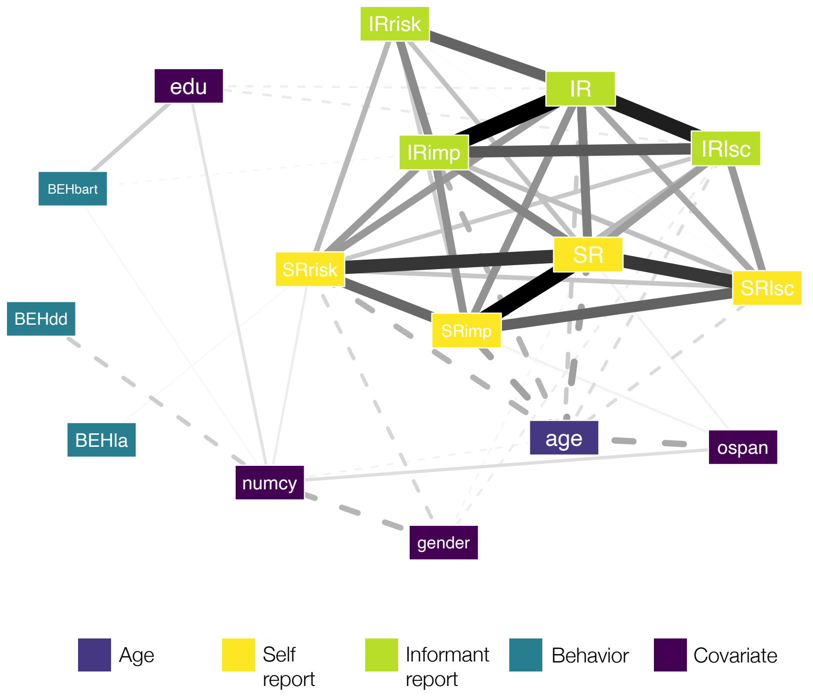

Supplementary Figure 11. Network plot of correlations between study variables collected in the extended sample (N=182). Variables were grouped by measure. Edge thickness is indicative of the strength of the correlation between two variables, with solid (dotted) edges representing positive (negative) associations. We only plotted correlations with absolute coefficients r ≥ 0.15. For labels, see Table 1.

Specification curve analysis

Main sample

The SCA results for the main sample (Supplementary Table 4) show converging negative effects of age on construct-specific and construct-independent composite scores based on self- and informant-report measures, but divergent age effects for behavioral measures.

Supplementary Table 4. Results of Specification Curve Analysis for the main sample (N = 148).

Number of specifications	480
Number of null age effects ($p > 0.05$)	188 (39.1%)
Number of significant age effects ($p \leq 0.05$)	292 (60.8%)
Number of positive age effects	20 (4.2%)
Number of negative age effects	272 (56.7%)
Median age effect size	-0.207
Number of shuffled samples with equal or more than 292 significant specifications	0/500
p-value of permutation test	0.00

Note: Rows may not add up to 100% due to rounding.

Extended sample

Out of the 176 specifications for the extended sample, 56 (31.8%) of specifications returned null effects (i.e., beta coefficients for age that were not significantly different from zero at p = 0.05), 4.6% returned significant positive age effects, and 63.4% returned significant negative age effects (Supplementary Table 5, Supplementary Figure 12). Ignoring the specific effects of unique specifications and aggregating across all 176 regression models, the median effect of age for the extended sample was -0.28. In other words, an increase in age by one SD was associated with a reduction in risk preference and related constructs of 0.28 SDs.

Supplementary Table 5. Results of Specification Curve Analysis for the extended (N = 182) sample.

Number of specifications	176
Number of null age effects (p > 0.05)	56 (31.8%)
Number of significant age effects (p ≤ 0.05)	120 (68.2%)
Number of positive age effects	8 (4.6%)
Number of negative age effects	112 (63.6%)
Median age effect size	-0.277
Number of shuffled samples with equal or more than 176 significant specifications	0/500
p-value of permutation test	0.00

Note: Rows may not add up to 100% due to rounding.

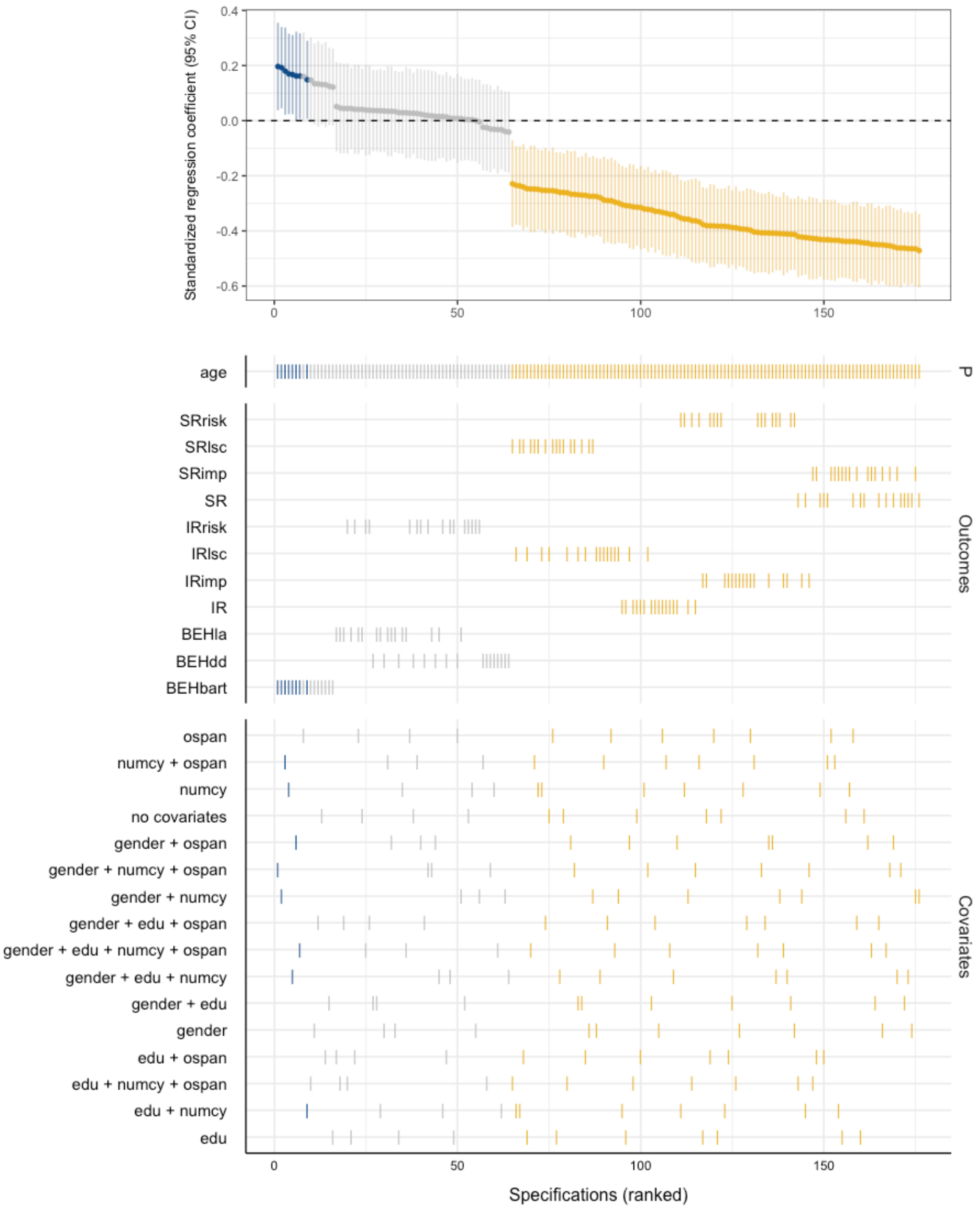

Supplementary Figure 12. Results of the SCA of age effects on risk preference and related constructs in the extended sample (N = 182). The top graph displays the standardized regression coefficients (95% CI) for age on varying operationalizations of risk preference, impulsivity, and low self-control. The specifications were ordered by effect size, from positive to negative. Tick marks in the lower panels describe the exact specifications, including which outcome and which covariate(s) were included in a given specification. P = predictor. Colors indicate significant ($p \leq 0.05$) positive (blue) and negative (orange) effects. For labels, see Table 1.

Permutation testing

Splitting the permutation results by effect direction revealed that none of the permutations resulted in more negative effects than observed in the original data (Supplementary Figure 13). In contrast, although most permutations yielded fewer positive effects than observed in the original data, a number of permutations yielded as many or more positive effects than observed in the original data. This pattern suggests that the negative effects are robust, but the positive effects may be less so. A similar pattern was observed for the extended sample (Supplementary Figure 14).

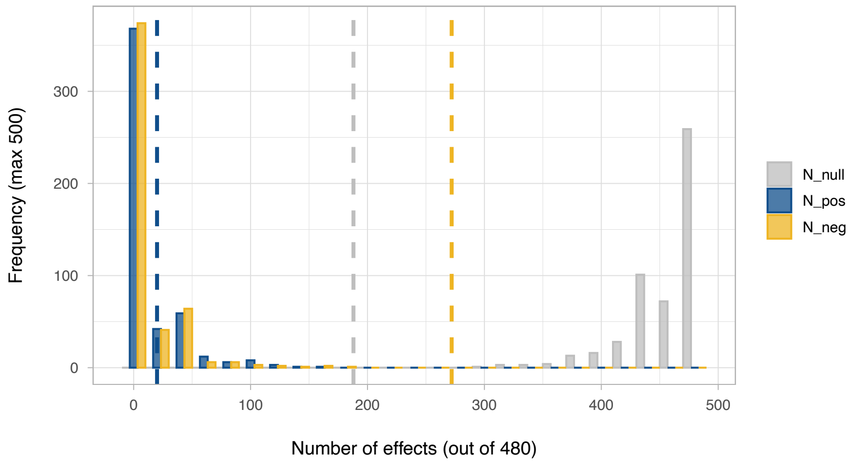

Supplementary Figure 13. Distribution of age effects in permutation analyses for the main sample. For the majority of permutations, significant negative (N_neg; beta < 0, p ≤ 0.05) and positive (N_pos; beta > 0, p ≤ 0.05) age effects were rarely obtained whereas null (N_null; p > 0.05) age effects were common across permutations. The x-axis intercept of the dashed lines shows the number of empirically observed null (gray), positive (blue), and negative (yellow) age effects in the original data.

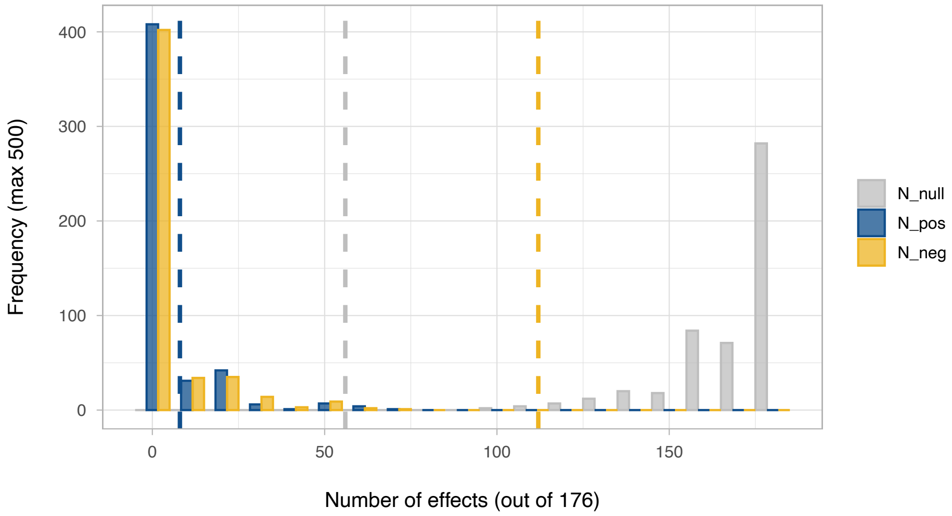

Supplementary Figure 14. Distribution of age effects in permutation analyses for the extended sample. For the majority of permutations, significant negative (N_neg; beta < 0, p ≤ 0.05) and positive (N_pos; beta > 0, p ≤ 0. 05) age effects were rarely obtained whereas null (N_null; p > 0.05) age effects were a common result across permutations. The x-axis intercept of the dashed lines shows the number of empirically observed age effects in the original data.

Exploratory analyses of informant-report indices

Based on the observed convergence of age effects for self- and informant-based indices, we sought to explore whether informant-reports capture idiosyncratic information about the study participant or converge due to methodological decisions related to item selection or response mode. For this purpose, we first examined whether the empirically observed correlations between self- and informant-based indices of risk preference, impulsivity, low self-control and across constructs were consistently higher than correlations based on random pairings of self- and informant-based indices. As shown in Supplementary Figure 15 (panel a), the empirically observed average correlations and their respective bootstrapped distributions were consistently different from and higher than average permutation-based correlations ($n = 500$ per construct). These results provide a first hint that informant-based indices are capturing idiosyncratic information associated with the target person (i.e., our study participants).

In a second step, we performed regression analyses to explore whether the absolute difference between self- and informant-based indices of risk preference, impulsivity, low self-control as well as across all constructs could be explained by target characteristics. In other words, we tested whether the magnitude of the difference between ratings of a person coming either from the person themselves or informants could be regressed onto the person's age, gender, education, numeracy, working memory, and, for the main sample, testosterone. Apart from using age as a predictor of the absolute difference between self- and informant-based indices, for consistency, we also included the covariates from the main analyses as predictors. Our results suggest that the calibration between the target person and informants was not influenced by the characteristics of the person (all $p > 0.05$, uncorrected) (Supplementary Table 6). We obtained comparable results for the extended sample (Supplementary Figure 15, panel b; Supplementary Table 7). These exploratory results suggest that informant-reports do capture unique information about a person and could thus be considered a promising elicitation method to capture individual differences.

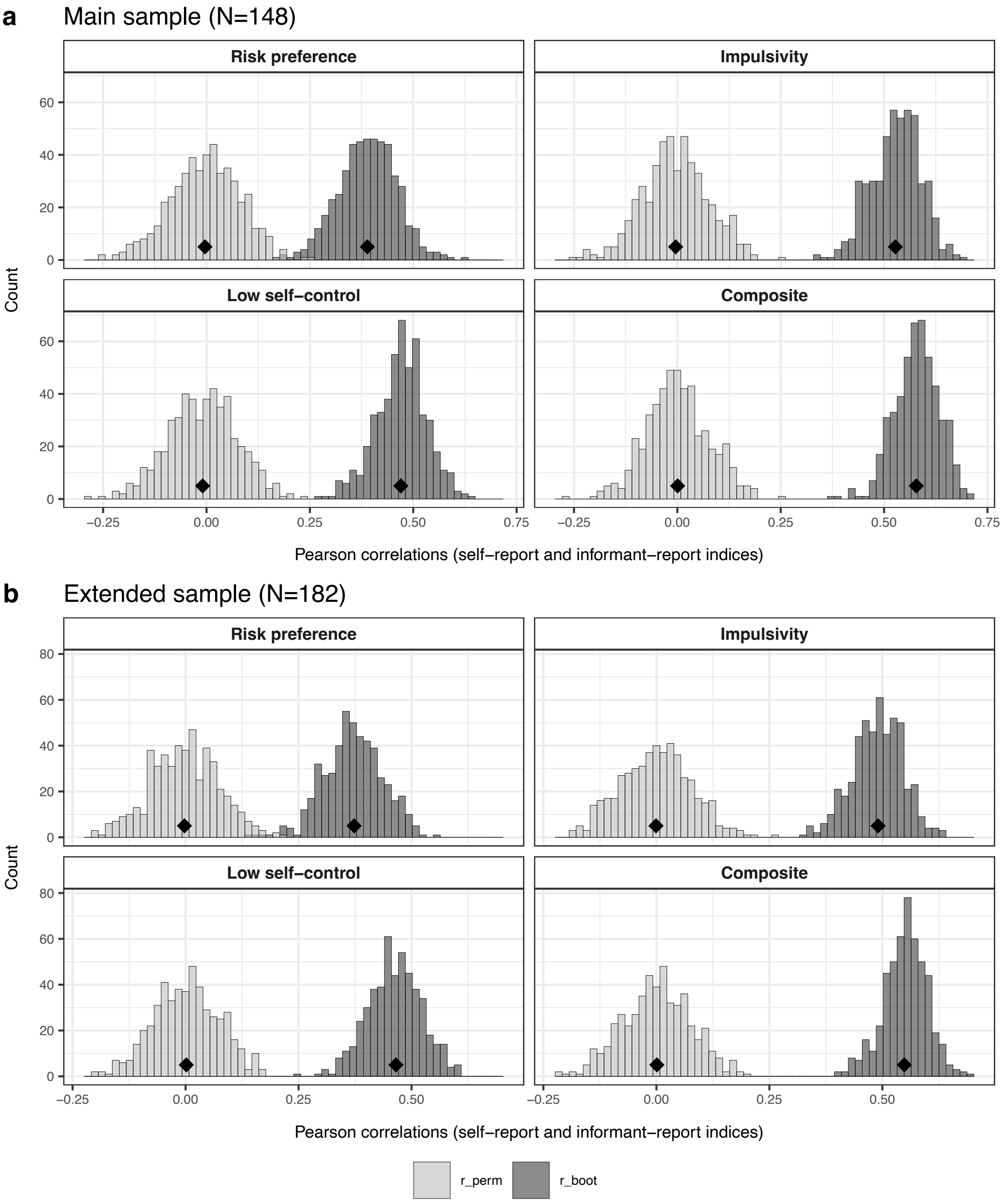

Supplementary Figure 15. Distribution of Pearson correlation coefficients for self- and informant-based indices based on true (dark) versus random (light) pairings, organized by construct. Black diamonds represent the mean correlation for random pairings and the empirically observed correlations between true pairings. (a) Main sample (N = 148). (b) Extended sample (N = 182). r_perm = distribution of permutation-based correlation coefficients for counterfactual, random pairings (n permutation-based samples = 500); r_boot = distribution of bootstrapped correlation coefficients for true pairings (n bootstrapped samples = 500).

Supplementary Table 6. Regression analyses of differences between self- and informant-based indices for the main sample (N = 148).

**Predictor**	**Δ Risk preference beta (SE)**	**Δ Impulsivity beta (SE)**	**Δ LSC beta (SE)**	**Δ All beta (SE)**
Age	-0.21 (0.26)	-0.21 (0.41)	0.47 (0.28)	-0.03 (0.71)
Gender	-0.34 (0.65)	-0.31 (1.00)	0.48 (0.67)	-0.60 (1.74)
Education	-0.40 (0.24)	-0.09 (0.37)	0.11 (0.25)	-0.22 (0.64)
Numeracy	-0.44 (0.27)	0.09 (0.42)	0.16 (0.28)	-0.14 (0.72)
Working memory	0.32 (0.26)	-0.27 (0.41)	0.11 (0.27)	-0.11 (0.71)
Testosterone	0.08 (0.31)	1.12 (0.48)	0.65 (0.32)	1.41 (0.83)
R^2	*0.06*	*0.09*	*0.06*	*0.05*

Note: beta = standardized beta coefficients; Δ = absolute difference between construct-specific composite indices based on self-report and informant-report; LSC = low self-control. All = composite score.

Supplementary Table 7. Regression analyses of differences between self- and informant-based indices for the extended sample (N = 182).

**Predictor**	**Δ Risk preference beta (SE)**	**Δ Impulsivity beta (SE)**	**Δ LSC beta (SE)**	**Δ All beta (SE)**
Age	-0.21 (0.23)	-0.30 (0.36)	0.15 (0.25)	-0.11 (0.62)
Gender	-0.37 (0.45)	-0.97 (0.72)	-0.36 (0.49)	-1.05 (1.24)
Education	-0.29 (0.21)	-0.13 (0.34)	0.23 (0.23)	-0.11 (0.58)
Numeracy	-0.25 (0.24)	0.18 (0.38)	0.23 (0.26)	0.25 (0.66)
Working memory	0.36 (0.23)	-0.35 (0.37)	0.03 (0.25)	-0.25 (0.63)
R^2	*0.04*	*0.02*	*0.02*	*0.01*

Note: b = standardized beta coefficients; Δ = absolute difference between construct-specific composite indices based on self-report and informant-report; LSC = low self-control. All = composite score.

References

Beauducel, A., Strobel, A., & Brocke, B. (2003). Psychometrische Eigenschaften und Normen einer deutschsprachigen Fassung der Sensation Seeking-Skalen, Form V. *Diagnostica*, *49*(2), 61–72. https://doi.org/10.1026/0012-1924.49.2.61

Bertrams, A., & Dickhäuser, O. (2009). Messung dispositioneller Selbstkontroll-Kapazität. *Diagnostica*, *55*(1), 2–10. https://doi.org/10.1026/0012-1924.55.1.2

Burr, D. A., Castrellon, J. J., Zald, D. H., & Samanez-Larkin, G. R. (2021). Emotion dynamics across adulthood in everyday life: Older adults are more emotionally stable and better at regulating desires. *Emotion*, *21*(3), 453–464.

Campbell, K. L., Lustig, C., & Hasher, L. (2020). Aging and inhibition: Introduction to the special issue. *Psychology and Aging*, *35*(5), 605–613. https://doi.org/10.1037/pag0000564

Carstensen, L. L. (2021). Socioemotional selectivity theory: The role of perceived endings in human motivation. *The Gerontologist*, *61*(8), 1188–1196. https://doi.org/10.1093/geront/gnab116

Carver, C. S., & White, T. L. (1994). Behavioral inhibition, behavioral activation, and affective responses to impending reward and punishment: The BIS/BAS Scales. *Journal of Personality and Social Psychology*, *67*, 319–333. https://doi.org/10.1037/0022-3514.67.2.319

Dalley, J. W., Everitt, B. J., & Robbins, T. W. (2011). Impulsivity, compulsivity, and top-down cognitive control. *Neuron*, *69*(4), 680–694. https://doi.org/10.1016/j.neuron.2011.01.020

Dalley, J. W., & Robbins, T. W. (2017). Fractionating impulsivity: Neuropsychiatric implications. *Nature Reviews Neuroscience*, *18*(3), 158–171. https://doi.org/10.1038/nrn.2017.8

Dariotis, J. K., Chen, F. R., & Granger, D. A. (2016). Latent trait testosterone among 18-24 year olds: Methodological considerations and risk associations. *Psychoneuroendocrinology*, *67*, 1–9. https://doi.org/10.1016/j.psyneuen.2016.01.019

De Groot, K. (2020). Burst beliefs – Methodological problems in the balloon analogue risk task and implications for its use. *Journal of Trial and Error*, *1*(1), 43–51. https://doi.org/10.36850/mr1

Depping, M. K., & Freund, A. M. (2011). Normal aging and decision making: The role of motivation. *Human Development*, *54*(6), 349–367. https://doi.org/10.1159/000334396

Desikan, R. S., Ségonne, F., Fischl, B., Quinn, B. T., Dickerson, B. C., Blacker, D., Buckner, R. L., Dale, A. M., Maguire, R. P., Hyman, B. T., Albert, M. S., & Killiany, R. J. (2006). An automated labeling system for subdividing the human cerebral cortex on MRI scans into gyral based regions of interest. *NeuroImage*, *31*(3), 968–980. https://doi.org/10.1016/j.neuroimage.2006.01.021

Duckworth, A. L., Peterson, C., Matthews, M. D., & Kelly, D. R. (2007). Grit: Perseverance and passion for long-term goals. *Journal of Personality and Social Psychology*, *92*(6), 1087–1101. https://doi.org/10.1037/0022-3514.92.6.1087

Duckworth, A. L., & Steinberg, L. (2015). Unpacking Self-Control. *Child Development Perspectives*, *9*(1), 32–37. https://doi.org/10.1111/cdep.12107

Düzel, E., Bunzeck, N., Guitart-Masip, M., & Düzel, S. (2010). NOvelty-related Motivation of Anticipation and exploration by Dopamine (NOMAD): Implications for healthy aging. *Neuroscience & Biobehavioral Reviews*, *34*(5), 660–669. https://doi.org/10.1016/j.neubiorev.2009.08.006

Eisenberg, I. W., Bissett, P. G., Enkavi, A. Z., Li, J., MacKinnon, D. P., Marsch, L. A., & Poldrack, R. A. (2019). Uncovering the structure of self-regulation through data-

driven ontology discovery. *Nature Communications*, *10*(1), 2319. https://doi.org/10.1038/s41467-019-10301-1

Eppinger, B., Nystrom, L. E., & Cohen, J. D. (2012). Reduced sensitivity to immediate reward during decision-making in older than younger adults. *PLoS ONE*, *7*(5), 10.

Epskamp, S., Cramer, A. O. J., Waldorp, L. J., Schmittmann, V. D., & Borsboom, D. (2012). Qgraph: Network visualizations of relationships in psychometric data. *Journal of Statistical Software*, *48*(4). https://doi.org/10.18637/jss.v048.i04

Fleckenstein, J., Schmidt, F. T. C., & Möller, J. (2014). Wer hat Biss? Beharrlichkeit und beständiges Interesse von Lehramtsstudierenden. Eine deutsche Adaption der 12-Item Grit Scale. *Psychologie in Erziehung Und Unterricht*, *61*, 281–286. https://doi.org/10.2378/peu2014.art

Frazier, I., Lighthall, N. R., Horta, M., Perez, E., & Ebner, N. C. (2019). CISDA: Changes in Integration for Social Decisions in Aging. *Wiley Interdisciplinary Reviews: Cognitive Science*, *10*(3), 1–15. https://doi.org/10.1002/wcs.1490

Frey, R., Pedroni, A., Mata, R., Rieskamp, J., & Hertwig, R. (2017). Risk preference shares the psychometric structure of major psychological traits. *Science Advances*, *3*(10), 1–13. https://doi.org/10.1126/sciadv.1701381

Frey, R., Richter, D., Schupp, J., Hertwig, R., & Mata, R. (2021). Identifying robust correlates of risk preference: A systematic approach using specification curve analysis. *Journal of Personality and Social Psychology*, *120*(2), 538–557. https://doi.org/10.1037/pspp0000287

Grasmick, H. G., Tittle, C. R., Bursik, R. J., & Arneklev, B. J. (1993). Testing the core empirical implications of Gottfredson and Hirschi's general theory of crime. *Journal of Research in Crime and Delinquency*, *30*(1), 5–29. https://doi.org/10.1177/0022427893030001002

Harden, K. P., Mann, F. D., Grotzinger, A. D., Patterson, M. W., Steinberg, L., Tackett, J. L., & Tucker-Drob, E. M. (2018). Developmental differences in reward sensitivity and sensation seeking in adolescence: Testing sex-specific associations with gonadal hormones and pubertal development. *Journal of Personality and Social Psychology*, *115*(1), 161–178. https://doi.org/10.1037/pspp0000172

Helfinstein, S. M., Schonberg, T., Congdon, E., Karlsgodt, K. H., Mumford, J. A., Sabb, F. W., Cannon, T. D., London, E. D., Bilder, R. M., & Poldrack, R. A. (2014). Predicting risky choices from brain activity patterns. *Proceedings of the National Academy of Sciences of the United States of America*, *111*(7), 2470–2475. https://doi.org/10.1073/pnas.1321728111

Kurath, J., & Mata, R. (2018). Individual differences in risk taking and endogeneous levels of testosterone, estradiol, and cortisol: A systematic literature search and three independent meta-analyses. *Neuroscience and Biobehavioral Reviews*, *90*(October 2017), 428–446. https://doi.org/10.1016/j.neubiorev.2018.05.003

Lejuez, C. W., Read, J. P., Kahler, C. W., Richards, J. B., Ramsey, S. E., Stuart, G. L., Strong, D. R., & Brown, R. A. (2002). Evaluation of a behavioral measure of risk taking: The Balloon Analogue Risk Task (BART). *Journal of Experimental Psychology. Applied*, *8*(2), 75–84. https://doi.org/10.1037/1076-898X.8.2.75

MacKillop, J., Weafer, J., C. Gray, J., Oshri, A., Palmer, A., & de Wit, H. (2016). The latent structure of impulsivity: Impulsive choice, impulsive action, and impulsive personality traits. *Psychopharmacology*, *233*(18), 3361–3370. https://doi.org/10.1007/s00213-016-4372-0

Masur, P. K., & Scharkow, M. (2020). *specr: Conducting and Visualizing Specification Curve Analyses*.

Mata, R., Josef, A. K., Samanez-Larkin, G. R., & Hertwig, R. (2011). Age differences in risky choice: A meta-analysis. *Annals of the New York Academy of Sciences*, *1235*(1), 18–29. https://doi.org/10.1111/j.1749-6632.2011.06200.x

Mather, M., Mazar, N., Gorlick, M. A., Lighthall, N. R., Burgeno, J., Schoeke, A., & Ariely, D. (2012). Risk preferences and aging: The “certainty effect” in older adults’ decision making. *Psychology and Aging*, *27*(4), 801–816. https://doi.org/10.1037/a0030174

Mazur, J. E. (1987). An adjusting procedure for studying delayed reinforcement. In M. L. Commons, J. E. Mazur, J. A. Nevin, & H. Rachlin (Eds.), *Quantitative analysis of behavior: Vol. 5. The effect of delay and of intervening events on reinforcement value* (Vol. 5, pp. 55–73). Erlbaum.

McAbee, S. T., & Connelly, B. S. (2016). A multi-rater framework for studying personality: The Trait-Reputation-Identity Model. *Psychological Review*, *123*(5), 569–591. https://doi.org/10.1037/rev0000035.supp

McClure, S. M., Laibson, D. I., Loewenstein, G., & Cohen, J. D. (2004). Separate neural systems value immediate and delayed monetary rewards. *Science*, *306*(5695), 503–507. https://doi.org/10.1126/science.1100907

Millroth, P., Juslin, P., Winman, A., Nilsson, H., & Lindskog, M. (2020). Preference or ability: Exploring the relations between risk preference, personality, and cognitive abilities. *Journal of Behavioral Decision Making*, *December 2019*, 1–15. https://doi.org/10.1002/bdm.2171

Mishra, S., Barclay, P., & Sparks, A. (2016). The Relative State Model: Integrating need-based and ability-based pathways to risk-taking. *Personality and Social Psychology Review*, 1–23. https://doi.org/10.1177/1088868316644094

Moffitt, T. E., Arseneault, L., Belsky, D., Dickson, N., Hancox, R. J., Harrington, H., Houts, R., Poulton, R., Roberts, B. W., Ross, S., Sears, M. R., Thomson, W. M., & Caspi, A.

(2011). A gradient of childhood self-control predicts health, wealth, and public safety. *Proceedings of the National Academy of Sciences*, *108*(7), 2693–2698. https://doi.org/10.1073/pnas.1010076108

Olschewski, S., Rieskamp, J., & Scheibehenne, B. (2018). Taxing cognitive capacities reduces choice consistency rather than preference: A model-based test. *Journal of Experimental Psychology: General*, *147*(4), 462–484. https://doi.org/10.1037/xge0000403

Orben, A., & Przybylski, A. K. (2019). The association between adolescent well-being and digital technology use. *Nature Human Behaviour*, *3*(2), 173–182. https://doi.org/10.1038/s41562-018-0506-1

Park, H., Yang, J., Vassileva, J., & Ahn, W. Y. (2019). *Exponential Weight Updating Model: A novel computational model for the Balloon Analogue Risk Task.*

Patton, J. H., Stanford, M. S., & Barratt, E. S. (1995). Factor structure of the Barratt Impulsiveness Scale. *Journal of Clinical Psychology*, *51*(6), 768–774. http://homepages.se.edu/cvonbergen/files/2013/01/Factor-Structure-of-the-Barratt-Impulsiveness-Scale.pdf

Pleskac, T. J. (2008). Decision making and learning while taking sequential risks. *Journal of Experimental Psychology: Learning Memory and Cognition*, *34*(1), 167–185. https://doi.org/10.1037/0278-7393.34.1.167

Pleskac, T. J., & Wershbale, A. (2014). Making assessments while taking repeated risks: A pattern of multiple response pathways. *Journal of Experimental Psychology: General*, *143*(1), 142–162. https://doi.org/10.1037/a0031106

Poldrack, R. A., & Mumford, J. A. (2009). Independence in ROI analysis: Where is the voodoo? *Social Cognitive and Affective Neuroscience*, *4*, 208–213. https://doi.org/10.1093/scan/nsp011

Pollet, T. V., & van der Meij, L. (2017). To remove or not to remove: The Impact of outlier handling on significance testing in testosterone data. *Adaptive Human Behavior and Physiology*, *3*(1), 43–60. https://doi.org/10.1007/s40750-016-0050-z

Preuss, U. W., Rujescu, D., Giegling, I., Watzke, S., Koller, G., Zetzsche, T., Meisenzahl, E. M., Soyka, M., & Möller, H. J. (2008). Psychometrische Evaluation der deutschsprachigen Version der Barratt-Impulsiveness Skala. *Der Nervenarzt*, *79*, 305–319. https://link.springer.com/article/10.1007/s00115-007-2360-7

Rao, H., Korczykowski, M., Pluta, J., Hoang, A., & Detre, J. A. (2008). Neural correlates of voluntary and involuntary risk taking in the human brain: An fMRI study of the Balloon Analog Risk Task (BART). *NeuroImage*, *42*(2), 902–910. https://doi.org/10.1016/j.neuroimage.2008.05.046

Rohrer, J. M., Egloff, B., & Schmukle, S. C. (2017). Probing birth-order effects on narrow traits using specification-curve analysis. *Psychological Science*, *28*(12), 1821–1832. https://doi.org/10.1177/0956797617723726

Sacchet, M. D., & Knutson, B. (2013). Spatial smoothing systematically biases the localization of reward-related brain activity. *NeuroImage*, *66*, 270–277. https://doi.org/10.1016/j.neuroimage.2012.10.056

Samanez-Larkin, G. R., & Knutson, B. (2015). Decision making in the ageing brain: Changes in affective and motivational circuits. *Nature Reviews Neuroscience*, *16*(May). https://doi.org/10.1038/nrn3917

Samanez-Larkin, G. R., Mata, R., Radu, P. T., Ballard, I. C., Carstensen, L. L., & McClure, S. M. (2011). Age differences in striatal delay sensitivity during intertemporal choice in healthy adults. *Frontiers in Neuroscience*, *5*(NOV), 1–12. https://doi.org/10.3389/fnins.2011.00126

Schmidt, R. E., Gay, P., D'Acremont, M., & Van Der Linden, M. (2008). A German adaptation of the UPPS impulsive behavior scale: Psychometric properties and factor structure. *Swiss Journal of Psychology*, *67*(2), 107–112. https://doi.org/10.1024/1421-0185.67.2.107

Schmitz, F., Manske, K., Preckel, F., & Wilhelm, O. (2016). The multiple faces of risk-taking: Scoring alternatives for the Balloon-Analogue Risk Task. *European Journal of Psychological Assessment*, *32*(1), 17–38. https://doi.org/10.1027/1015-5759/a000335

Schonberg, T., Fox, C. R., Mumford, J. A., Congdon, E., Trepel, C., & Poldrack, R. A. (2012). Decreasing ventromedial prefrontal cortex activity during sequential risk-taking: An FMRI investigation of the balloon analog risk task. *Frontiers in Neuroscience*, *6*(June), 1–11. https://doi.org/10.3389/fnins.2012.00080

Schonberg, T., Fox, C. R., & Poldrack, R. A. (2011). Mind the gap: Bridging economic and naturalistic risk-taking with cognitive neuroscience. *Trends in Cognitive Sciences*, *15*(1), 11–19. https://doi.org/10.1016/j.tics.2010.10.002

Seaman, K. L., Abiodun, S., Fenn, Z., Samanez-Larkin, G. R., & Mata, R. (2022). Temporal discounting across adulthood: A systematic review and meta-analysis. *Psychology and Aging*, *37*(1), 111–124. https://doi.org/10.31234/osf.io/7ysxa

Seaman, K. L., Brooks, N., Karrer, T. M., Castrellon, J. J., Perkins, S. F., Dang, L. C., Hsu, M., Zald, D. H., & Samanez-Larkin, G. R. (2018). Subjective value representations during effort, probability and time discounting across adulthood. *Social Cognitive and Affective Neuroscience*, *13*(5), 449–459. https://doi.org/10.1093/scan/nsy021

Seipel, C. (2014). Deutsche Version der Self-Control Skala. In *Zusammenstellung sozialwissenschaftlicher Items und Skalen*. https://doi.org/10.6102/zis137

Shao, R., & Lee, T. M. C. (2014). Aging and risk taking: Toward an integration of cognitive, emotional, and neurobiological perspectives. *Neuroscience and Neuroeconomics*, *3*, 47–62. https://doi.org/10.2147/NAN.S35914

Simonsohn, U., Simmons, J. P., & Nelson, L. D. (2020). Specification curve analysis. *Nature Human Behaviour*, *4*(11), 1208–1214. https://doi.org/10.1038/s41562-020-0912-z

Steegen, S., Tuerlinckx, F., Gelman, A., & Vanpaemel, W. (2016). Increasing transparency through a multiverse analysis. *Perspectives on Psychological Science*, *11*(5), 702–712. https://doi.org/10.1177/1745691616658637

Steinberg, L., Albert, D., Cauffman, E., Banich, M., Graham, S., & Woolard, J. (2008). Age differences in sensation seeking and impulsivity as indexed by behavior and self-report: Evidence for a dual systems model. *Developmental Psychology*, *44*(6), 1764–1778. https://doi.org/10.1037/a0012955

Steiner, M. D., & Frey, R. (2021). Representative design in psychological assessment: A case study using the Balloon Analogue Risk Task (BART). *Journal of Experimental Psychology: General*, 1–24. https://doi.org/10.1037/xge0001036

Strobel, A., Beauducel, A., & Debener, S. (2001). Psychometrische und strukturelle Merkmale einer deutschsprachigen Version des BIS / BAS-Fragebogens. *Zeitschrift Für Differentielle Und Diagnostische Psychologie*, *22*(3), 216–227.

Tangney, J. P., Baumeister, R. F., & Boone, A. L. (2004). High self-control predicts good adjustment, less pathology, better grades, and interpersonal success. *Journal of Personality*, *72*(2), 271–324. https://onlinelibrary.wiley.com/doi/pdf/10.1111/j.0022-3506.2004.00263.x

Tannou, T., Magnin, E., Comte, A., Aubry, R., & Joubert, S. (2021). Neural activation in risky decision-making tasks in healthy older adults: A meta-analysis of fmri data. *Brain Sciences*, *11*(8). https://doi.org/10.3390/brainsci11081043

Tisdall, L., Frey, R., Horn, A., Ostwald, D., Horvath, L., Pedroni, A., Rieskamp, J., Blankenburg, F., Hertwig, R., & Mata, R. (2020). Brain-outcome associations for risk taking depend on the measures used to capture individual differences. *Frontiers in Behavioral Neuroscience*, *14*(November). https://doi.org/10.31234/osf.io/3sc9j

TNS Infratest Sozialforschung. (2014). SOEP 2014 – Erhebungsinstrumente 2014 (Welle 31) des Sozio-oekonomischen Panels: Personenfragebogen, Altstichproben. *SOEP Survey Papers 235: Series A*.

Tom, S. M., Fox, C. R., Trepel, C., & Poldrack, R. A. (2007). The neural basis of loss aversion in decision-making under risk. *Science*, *315*(5811), 515–518. https://doi.org/10.1126/science.1134239

Unsworth, N., Heitz, R. P., Schrock, J. C., & Engle, R. W. (2005). An automated version of the operation span task. *Behaviour Research Methods*, *37*(3), 498–505. https://link.springer.com/content/pdf/10.3758/bf03192720.pdf

van den Bos, W., Rodriguez, C. A., Schweitzer, J. B., & McClure, S. M. (2014). Connectivity strength of dissociable striatal tracts predict individual differences in temporal discounting. *Journal of Neuroscience*, *34*(31), 10298–10310. https://doi.org/10.1523/JNEUROSCI.4105-13.2014

van Ravenzwaaij, D., Dutilh, G., & Wagenmakers, E. J. (2011). Cognitive model decomposition of the BART: Assessment and application. *Journal of Mathematical Psychology*, *55*(1), 94–105. https://doi.org/10.1016/j.jmp.2010.08.010

Wallsten, T. S., Pleskac, T. J., & Lejuez, C. W. (2005). Modeling behavior in a clinically diagnostic sequential risk-taking task. *Psychological Review*, *112*(4), 862–880. https://doi.org/10.1037/0033-295X.112.4.862

Weber, E. U., Blais, A.-R., & Betz, N. E. (2002). A domain-specific risk-attitude scale: Measuring risk perceptions and risk behaviors. *Journal of Behavioral Decision Making*, *15*(August), 263–290. https://doi.org/10.1002/bdm.414

Weller, J. A., Dieckmann, N. F., Tusler, M., Mertz, C. K., Burns, W. J., & Peters, E. (2013). Development and testing of an abbreviated numeracy scale: A Rasch analysis approach. *Journal of Behavioral Decision Making*, *26*(2), 198–212. https://doi.org/10.1002/bdm.1751

Whiteside, S. P., & Lynam, D. R. (2001). The five factor model and impulsivity: Using a structural model of personality to understand impulsivity. *Personality and Individual Differences*, *30*(4), 669–689. https://doi.org/10.1016/S0191-8869(00)00064-7

Wulff, D. U., & van den Bos, W. (2018). Modeling choices in delay discounting. *Psychological Science*, *29*(11), 1890–1894. https://doi.org/10.1177/0956797616664342

Zank, H. (2010). On probabilities and loss aversion. *Theory and Decision*, *68*(3), 243–261. https://doi.org/10.1007/s11238-008-9117-z

Zimmermann, E., Budowski, M., Gabadinho, A., Scherpenzeel, A., Tillmann, R., & Wernli, B. (2003). The Swiss household panel survey: A multidimensional database for monitoring social change. In D. Joye, I. Renschler, & F. Hainard (Eds.), *Social change and social reporting* (pp. 137–156). UNESCO and SIDOS.

Zuckerman, M., Eysenck, S. B., & Eysenck, H. J. (1978). Sensation seeking in England and America: Cross-cultural, age, and sex comparisons. *Journal of Consulting and Clinical Psychology*, *46*(1), 139–149. https://doi.org/10.1037/0022-006X.46.1.139
